# Developing Cheap but Useful Machine Learning-Based Models for Investigating High-Entropy Alloy Catalysts

**DOI:** 10.1021/acs.langmuir.3c03401

**Published:** 2024-02-05

**Authors:** Chenghan Sun, Rajat Goel, Ambarish R. Kulkarni

**Affiliations:** Department of Chemical Engineering, University of California, Davis, California 95616, United States

## Abstract

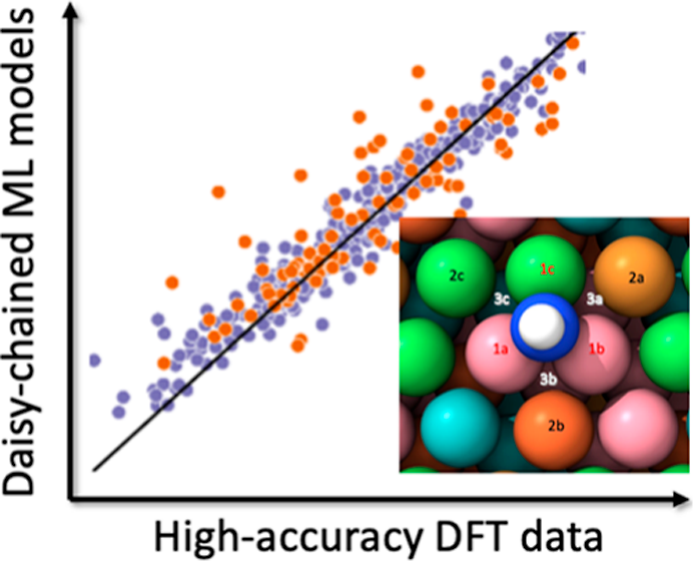

This work aims to address the challenge of developing interpretable ML-based models when access to large-scale computational resources is limited. Using CoMoFeNiCu high-entropy alloy catalysts as an example, we present a cost-effective workflow that synergistically combines descriptor-based approaches, machine learning-based force fields, and low-cost density functional theory (DFT) calculations to predict high-quality adsorption energies for H, N, and NH_*x*_ (*x* = 1, 2, and 3) adsorbates. This is achieved using three specific modifications to typical DFT workflows including: (1) using a sequential optimization protocol, (2) developing a new geometry-based descriptor, and (3) repurposing the already-available low-cost DFT optimization trajectories to develop a ML-FF. Taken together, this study illustrates how cost-effective DFT calculations and appropriately designed descriptors can be used to develop cheap but useful models for predicting high-quality adsorption energies at significantly lower computational costs. We anticipate that this resource-efficient philosophy may be broadly relevant to the larger surface catalysis community.

## Introduction

The development of machine learning (ML) models has revolutionized the computational catalysis community in several different ways.^1−3^ One powerful application of these techniques is the use of ML to predict adsorption energies of key reaction intermediates. These adsorption energies serve as descriptors to accelerate theory-guided design of promising catalysts.^4,5^ Here, ML is used to circumvent the computational costs associated with exhaustive density functional theory (DFT) calculations. Specifically, instead of using brute force DFT to sample the entire composition-space (e.g., for bimetallic and multimetallic alloys) and configuration-space (e.g., various facets and binding sites) of interest, a smaller database of DFT calculations (i.e., the training data set) is used to develop a surrogate predictive model. Once validated, the resulting ML model is then used explore a much broader catalyst design space that is generally inaccessible with DFT. This design philosophy has motivated the development of several large catalysis-focused databases (e.g., the open catalyst database^6,7^), open source software,^8^ and a growing list of ML models and descriptors.^9−15^ The recent advances, challenges, and opportunities in this field have been previously reviewed.^16−19^

Assuming an idealized scenario where the computationally identified active site motif is experimentally realizable and stable under the reaction conditions, which may not always be true, the overall success of the aforementioned screening studies depends on the accuracy and reliability of the surrogate ML model.^20^ Thus, it becomes necessary to ensure that the initial training data set is sufficiently large and diverse to capture the underlying complexity of the entire phase space. With the advent of new descriptors^21−24^ and highly complex ML models,^25−27^ in our opinion, the development of “universal” models of adsorption energies for surface-mediated reactions can often be limited by the access to high-performance computing (HPC) resources. Specifically, the computational costs are associated with creating a large database of DFT calculations and training ML models; the latter step is usually accelerated by using GPUs. For example, the OC22 database, comprising more than 62,000 DFT relaxations, required approximately 10 million individual single-point energy (SPE) calculations. This data set, which utilized the PBE + U method (similar to the Materials Project),^8,28^ required over 240 million core-hours.^7^

However, we note that these scales of HPC resources are often not available to academic research groups. These observations echo similar trends within the ML community. For example, while the development of large language models (e.g., ChatGPT, LLAMA, Bard, etc.) is led by commercial entities, the academic ML community tends to focus on developing new algorithms, demonstrating computational frameworks and workflows, and fine-tuning the existing open source models. Thus, even within the context of catalysis science, it is plausible that compute-intensive aspects of the data-generation and model development will stay (or become) outside the purview of university-led research. Thus, in this study, we focus on exploring strategies to develop interpretable ML models while being mindful of the associated computational costs.

As the first step toward this goal, here, we demonstrate how low-cost DFT calculations (performed using four-layer constrained slabs, 300 eV energy cutoff, 2 × 2 × 1 *k*-points) can be used to predict adsorption energies at a significantly higher accuracy (i.e., four-layer slab with top two layers allowed to relax, 700 eV cutoff, 3 × 3 × 1 *k*-points). We illustrate the efficacy of this approach by investigating the catalytic properties of CoMoFeNiCu high-entropy alloy (HEA) catalysts for the ammonia decomposition reaction. The chosen system is motivated by the experimental results of Xie et al.^29^ In this study, the authors present NH_3_ decomposition rates for a range of Co/Mo compositions (i.e., 15/55% to 55/15%). Compared to Ru-based catalysts, the authors showed improved catalytic activities for the ammonia decomposition reaction, which was attributed to the continuously varying alloy composition. More recently, this reaction has been computationally investigated by Saidi et al.^14^ The authors used a convolutional neural network (CNN) model to predict that a 25/45% Co/Mo composition will result in higher rates for ammonia decomposition. The Saidi et al. data set consists of 19,911 DFT calculations using a 300 eV energy cutoff, 3 × 3 × 1 *k*-points, and the PBE functional. The CNN model, which uses a combination of element-specific features, d-band properties, and geometric parameters as fingerprints, results in mean absolute errors (MAE) of 0.05 eV for the binding energies. We note that the plane wave cutoff used by the authors (i.e., 300 eV) is lower than the typical values (usually 400 eV or higher) used within the field. In light of this discussion, our goal is to develop predictive and interpretable models that can achieve higher numerical accuracies (e.g., using a 700 eV cutoff) but require lower computational costs.

Our approach is facilitated by three modifications to the typical workflows used within the field. First, instead of relying on a direct single-step DFT optimization at the required computational accuracy, we use a sequential multistep optimization protocol, which provides useful information at each step. This approach results in faster convergence at a lower computational cost. Second, we utilize a new geometry-based descriptor to predict adsorption energies. Specifically, the Generalized Local Structure-Sensitive (GLaSS) descriptor uses DFT-optimized structures as inputs and captures geometric proprieties such as interatomic distances, angles, and dihedrals. This work is inspired by the recent studies by Batchelor et al.^11^ and Pedersen et al.,^30^ where the highly symmetric site motifs of HEA surfaces are represented using combinatorial linear relationships. Instead, the proposed GLaSS descriptor does not require any assumptions about the specific high-symmetry binding sites and, thus, can be effortlessly extended to arbitrary binding sites. As shown later, the resulting MAEs (obtained using the GLaSS descriptor) are comparable to or lower than the state-of-the-art geometric descriptors, e.g., Smooth Overlap of Atomic Positions (SOAP).^21,23,31^ As the third modification, we investigate the potential of utilizing low-quality DFT data (denoted as ΔE_DFT_^cons,low^) to predict higher accuracy results (denoted as Δ*E*_DFT_^relax,high^). Thus, in addition to the geometric information provided by the GLaSS descriptor, the inclusion of ΔE_DFT_^cons,low^ provides information about the binding energies, which also improves the interpretability of the model.

**Figure 1:**
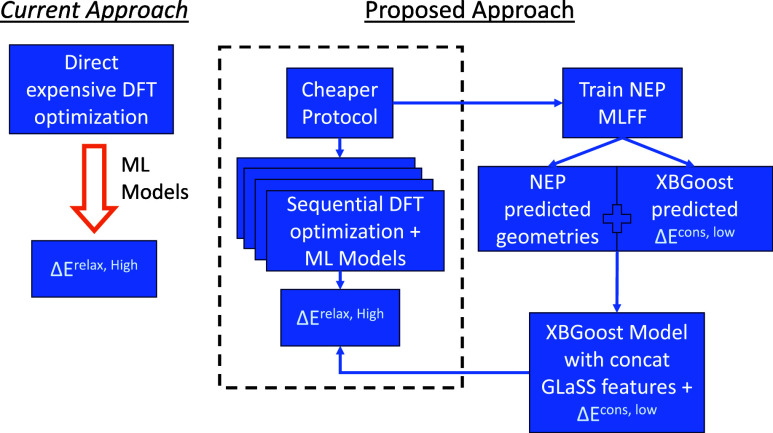
Schematic overview of the proposed daisy chained model used to predict high-accuracy DFT adsorption energies (Δ*E*_DFT_^relax,high^). This approach, which requires less expensive DFT calculations, provides comparable accuracies at lower costs.

A key limitation of the GLaSS approach, however, is that it requires the use of DFT-optimized geometries as inputs to the ML model. Although this results in accurate predictions, using DFT-optimized structures to predict DFT-binding energies defeats the entire purpose of developing an ML model. As a potential solution, we show that the trajectories obtained during the Δ*E*_DFT_^cons,low^ calculation can be repurposed to overcome this bottleneck. Specifically, we show that the already available DFT data from low-cost geometry relaxations can be used to develop a machine learning force field (ML-FF). This ML-FF, in turn, serves as a DFT-free approach that provides reasonably accurate estimates of the DFT-optimized binding geometries. Combined with the GLaSS descriptor, the ML-FF-derived geometries can then be used to calculate Δ*E*_DFT_^cons,low^ and *E*_DFT_^relax,high^. This sequential workflow is schematically illustrated in Figure 1. Impressively, the final daisy chained DFT-free model shows reasonable MAEs (max of 187 meV/atom) across five adsorbates (H, N, NH, NH_2_, and NH_3_) for CoMoFeNiCu HEA. We note that the proposed refinement strategy has been discussed previously by Chen et al.^32^

In summary, this study illustrates a potential strategy for generating high-quality DFT data using fewer computational resources while also reusing low-quality data that is already available during a geometry optimization. Although this work has focused on investigating the CoMoFeNiCu HEA catalysts for a specific reaction (i.e., ammonia decomposition), we anticipate that an analogous approach can be applied for other HEA-catalyzed reactions and, subsequently, to other types of catalysts.

## Methods

### DFT Calculations

The CoMoFeNiCu HEAs catalysts are modeled as an ideal FCC(111) surface using 3 × 3 × 4 slab model. The Co/Mo elemental ratio is varied from 15/55% to 55/15% in 10% increments, resulting in five distinct HEA compositions. The fraction of Fe, Ni, and Cu is kept fixed at 10% to reflect the HEA systems studied by Xie et al. For each of the five Co/Mo compositions, we created 100 unique surfaces with random arrangements of the five elements. Following Batchelor et al.,^11^ a composition-dependent lattice constant is used. The simulated lattice constants ranged from 3.628 Å for Co_55_Mo_15_FeNiCu to 3.766 Å for Co_15_Mo_55_FeNiCu. These values are in close agreement with the experimental lattice constant of 3.74 Å.^29^ Five different adsorbates relevant to the NH_3_ decomposition reaction are considered: H, N, NH, NH_2_, and NH_3_. Additionally, as several different binding sites with varying local environments are possible for a given surface [e.g., 9 on-top sites, 27 bridge sites, and 18 hollow sites for a 3 × 3 fcc(111) facet], we obtain ca. 135,000 total possible sites for 100 HEA surfaces across five Co/Mo compositions. This ensemble of possible unique binding sites further emphasizes the need of developing cheaper ML alternatives instead of using brute force DFT calculations.

To capture the diversity of possible local environments, we carefully selected one to two binding sites from each surface. As a result, a total of 2643 (i.e., 480 sites for H, 466 for N, 586 for NH, 570 for NH_2_, and 541 for NH_3_) sites are sampled for DFT geometry optimizations. This sampling approach ensures that the training data set is representative of the desired exploration space. As described previously, two separate sequential optimizations are performed for each of the 2643 sites. Specifically, while all the metal atoms are held fixed for the constrained surfaces (i.e., at 300 eV, 2 × 2 × 1 *k*-points), the top two layers are allowed to relax for the high-accuracy optimization (i.e., at 700 eV, 3 × 3 × 1 *k*-points). Taken together, the CoMoFeNiCu HEA database consists of 5286 adsorption energies and geometries. The high-accuracy adsorption energies (Δ*E*_DFT_^relax,high^) and the low DFT-accuracy adsorption energies Δ*E*_DFT_^cons,low^ are calculated as
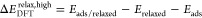
1
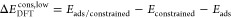
2where *E*_ads/relaxed_ and *E*_ads/constrained_ represent the energies of relaxed and constrained adsorbate-bound surfaces, respectively; *E*_relaxed_ and *E*_constrained_ represent the energies of bare relaxed and constrained surfaces, respectively; and *E*_ads_ represents the DFT energy of each adsorbate in the gas phase. As entropic contributions are not included, here, we limit our discussion to the DFT-calculated binding energies.

The Vienna ab initio simulation package (VASP)^33−35^ was used for all DFT calculations. The revised PBE from Hammer et al.^36^ (RPBE) exchange–correlation functional was used; dispersion corrections were not included. The conjugate gradient algorithm was used for geometry optimizations. The DFT optimizations were terminated when the forces on each atom were below the 0.05 eV/Å threshold. For the sequential DFT optimization protocol, we used a range of energy cutoffs (i.e., 300:100:700 eV) and *k*-meshes (i.e., 2 × 2 × 1 and 3 × 3 × 1). Specifically, the sequential optimization consisted of (30 optimization steps at 300 eV, 2 × 2 × 1), (30 steps at 400 eV, 2 × 2 × 1), (10 steps at 400 eV, 3 × 3 × 1), (10 steps at 500 eV, 3 × 3 × 1), (10 steps at 600 eV, 3 × 3 × 1), and final convergence at (700 eV, 3 × 3 × 1). Note that our ML models aim to predict the high-accuracy adsorption energies (i.e., Δ*E*_DFT_^relax,high^), which are obtained using relaxed surfaces at 700 eV energy cutoff and 3 × 3 × 1 *k*-points. The entire DFT data set is available in the Supporting Information.

### ML-FF Training

A staged downsampling strategy was used to obtain configurations for ML-FF training using the low-cost, low-accuracy geometry optimizations. For example, if a geometry optimization (i.e., at 300 eV, 2 × 2 × 1 *k*-points) required 300 total ionic steps, then every configuration from the first 50 ionic steps was included in the training. Subsequently, the sampling frequency was reduced such that every other configuration was sampled for steps 51–100, 1 in 3 for steps 101–150, and 1 in 4 for steps 151–200, and so on. This approach increases the diversity of the configurations used in the ML-FF development while reducing the inclusion of almost similar structures that are often encountered during the final stages of DFT geometry optimization.

The above data set was used to develop a ML-FF using the neuroevolution potential (NEP) interatomic potential, as implemented in the Graphics Processing Units Molecular Dynamics (GPUMD) package (version 3.8).^37,38^ The performance of the NEP potential is strongly dependent on several key parameters. These include (1) Rc_ut_^radial^ and Rc_ut_^angular^, which determine the effective range of interatomic interactions and (2) λ_e_ and λ_f_, which control the relative contributions of the force and energy terms to the NEP loss function. As summarized in Table 1, these parameters were chosen by using a series of hyperparameter sensitivity studies.

**Table 1:** Optimal Hyper-Parameters for the NEP-ML-FF

*R*_cut_^radial^ (Å)	*R*_cut_^angular^ (Å)	*N*_bas_^radial^	*N*_bas_^angular^	*n*_max_^radial^
7.5	7.5	12	12	8

*n*_max_^angular^	*l*_3b_^max^/*l*_4b_^max^/*l*_5b_^max^	*N*_neu_	λ_e_	λ_f_
8	4/2/0	50	0.01	0.99

λ_1_	λ_2_	*N*_bat_	*N*_pop_	*N*_gen_
–1 (default)	–1 (default)	1000	50	100,000

As the NEP ML-FF is used as a surrogate model for constructing the geometry-based GLaSS descriptor, we use mean square deviations (MSDs) to quantify the efficacy of the ML-FF. Here, the MSDs between pairs of configurations optimized via DFT and NEP-ML-FF are calculated as follows
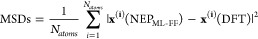
3

As the atoms within the HEA surfaces remained fixed in the low-accuracy optimization, the MSD is calculated using only the positions of the N atom of each adsorbate. The vectors **x**^(**i**)^(DFT) and **x**^(**i**)^(NEP_ML-FF_) denote the positions of the i-th atoms subsequent to DFT and NEP-ML-FF optimizations, respectively.

### XGBoost and Optuna

We utilized XGBoost in conjunction with Optuna (for hyperparameter optimization (HPO)) to develop the necessary ML models. For the learning task parameters, we used the default regression with squared loss as the learning objective and MAE as the evaluation metric. We considered two booster types, gbtree and dart, excluding the gblinear booster due to its lower performance. The HPO for tree boosters focused on general parameters such as the learning rate (eta), minimum loss reduction for further partitioning (gamma), maximum tree depth (max_depth), and minimum sum of instance weight (min_child_weight). The tree growing policy was chosen to be either depthwise or lossguide. For the dart booster, additional hyperparameters were considered, including the type of sampling algorithm (sample_type), normalization algorithm (normalize_type), dropout fraction (rate_drop), and dropout skipping probability (skip_drop). Furthermore, regularization parameters such as L2 and L1 regularization terms (lambda and alpha), the subsample ratio for training data (subsample), and the sampling ratio per tree (colsample_bytree) were included to prevent overfitting.

Enabling the Optuna HPO is a straightforward process. Our objective was to minimize the average MAE obtained through fivefold cross-validation (CV) in XGBoost. The number of boosting iterations ranged from 50 to 70, and the number of early stopping rounds varied from 5 to 10 to address overfitting issues. To further limit overfitting, we incorporated XGBoostPruningCallback integrated with Optuna into the CV process. The hyperparameters of XGBoost that were subject to optimization were suggested using a trial object with some hyperparameters (eta, gamma, lambda, alpha, rate_drop, and skip_drop) sampled from the logarithmic domain. A study object was created to execute the HPO, which involved conducting 300 trials, each representing the evaluation of an objective function. We also used the Optuna-integrated MedianPruner to perform pruning if a trial’s best intermediate result was inferior to the median of intermediate results from previous trials at the same step.

The training loss curves for all the XGBoost models using the GLaSS^relax,high^ descriptor and the GLaSS_NEP_^cons,low^ + Δ*E*_ML_^cons,low^ descriptor are plotted and presented in Figures S9 and 10, respectively. These curves provide a visual representation of the training process.

## Results and Discussion

**Figure 2:**
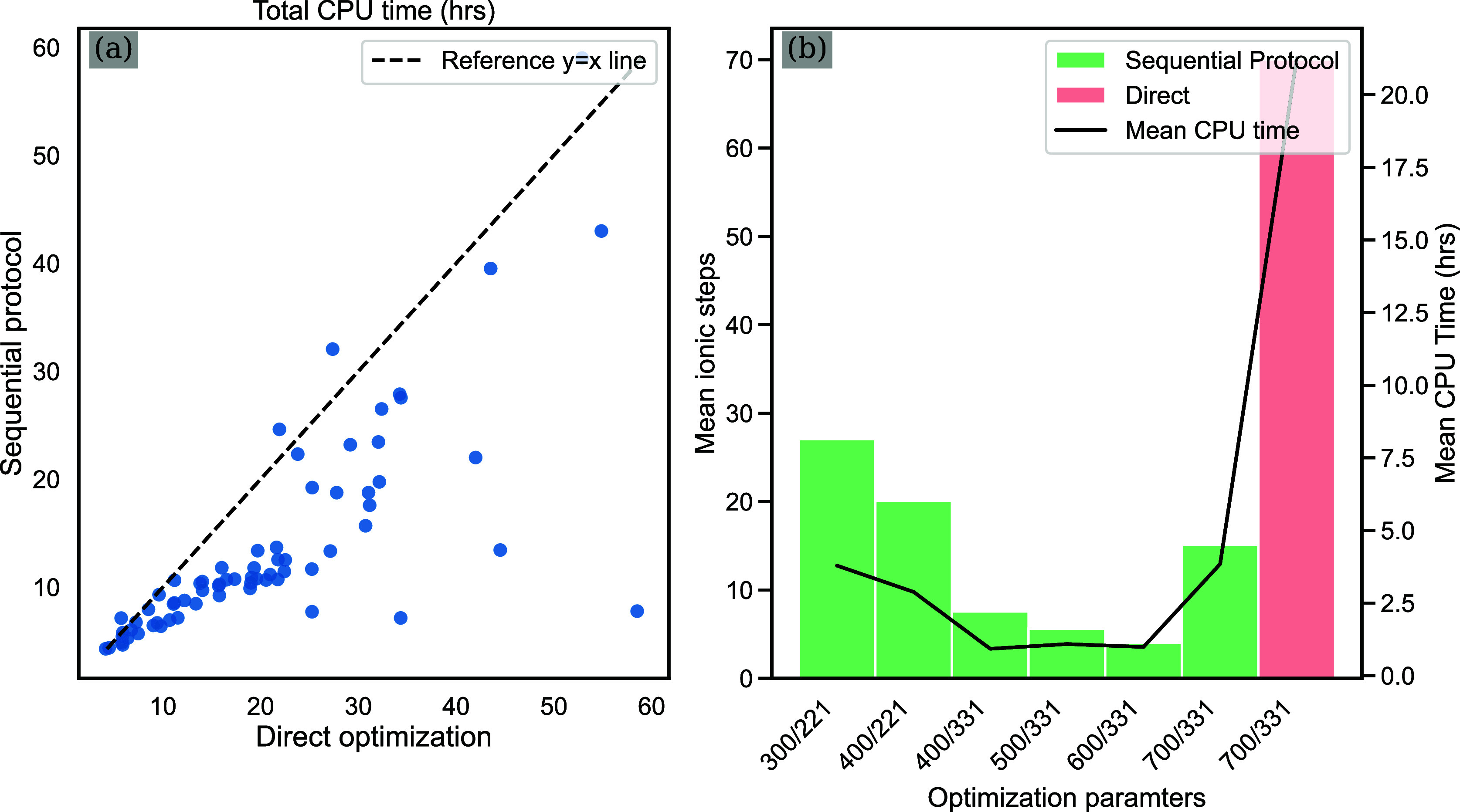
CPU cost comparison of sequential DFT optimization protocol versus the direct DFT optimization protocol. (a) CPU cost comparison (in hours) for two optimization protocols performed on the same subset of randomly selected relaxed CoMoFeNiCu sites across five adsorbates. (b) Average number of ionic steps and associated CPU time costs between two optimization protocols.

We begin the discussion by examining the costs of generating high-quality DFT data for the chosen CoMoFeNiCu HEAs system. Here, the high-quality binding energies, denoted as Δ*E*_DFT_^relax,high^, are obtained using the revised Perdew–Burke–Ernzerhof (RPBE)^36^ functional, 700 eV energy cutoff, and 3 × 3 × 1 *k*-points (denoted as 700/331). For a subset of 125 randomly selected relaxed HEA sites equally across five adsorbates, Figure 2a compares the computational cost of the sequential DFT optimization protocol (as shown in Figure 2b) to the one-step direct optimization protocol. Within the sequential optimization protocol, energy cutoffs are gradually increased from 300 to 700 eV in intervals of 100 eV. The 2 × 2 × 1 *k*-point mesh is used for 300 and 400 eV cutoffs, while the 3 × 3 × 1 *k*-point mesh is adopted for the remaining stages. On average, we observe that the sequential optimization is 64% faster than the direct optimization protocol and requires fewer number of ionic steps at the desired DFT parameters. As shown in Figure 2b, this is because a large fraction of the ionic steps are performed at lower accuracy settings that require a lower per self-consistent field step cost. On average, the low-quality adsorption energies calculations (obtained using constrained surfaces with 300 eV cutoff and 2 × 2 × 1 *k*-points) are 4.26 times faster than an analogous 700/331 calculations for the same geometry. Thus, the sequential protocol depicted in Figure 2b was employed to obtain Δ*E*_DFT_^relax,high^ until convergence to a DFT accuracy of 700/331. In parallel, direct optimizations carried out at an accuracy of 300/221 were utilized for the computation of Δ*E*_DFT_^cons,low^. Both of these two sets of low-accuracy and high-accuracy DFT optimizations are performed across the same training data set, encompassing a total of 2109 distinct sites. More details about CoMoFeNiCu HEAs surface slab modeling and corresponding DFT optimizations are provided in the Methods section.

**Figure 3:**
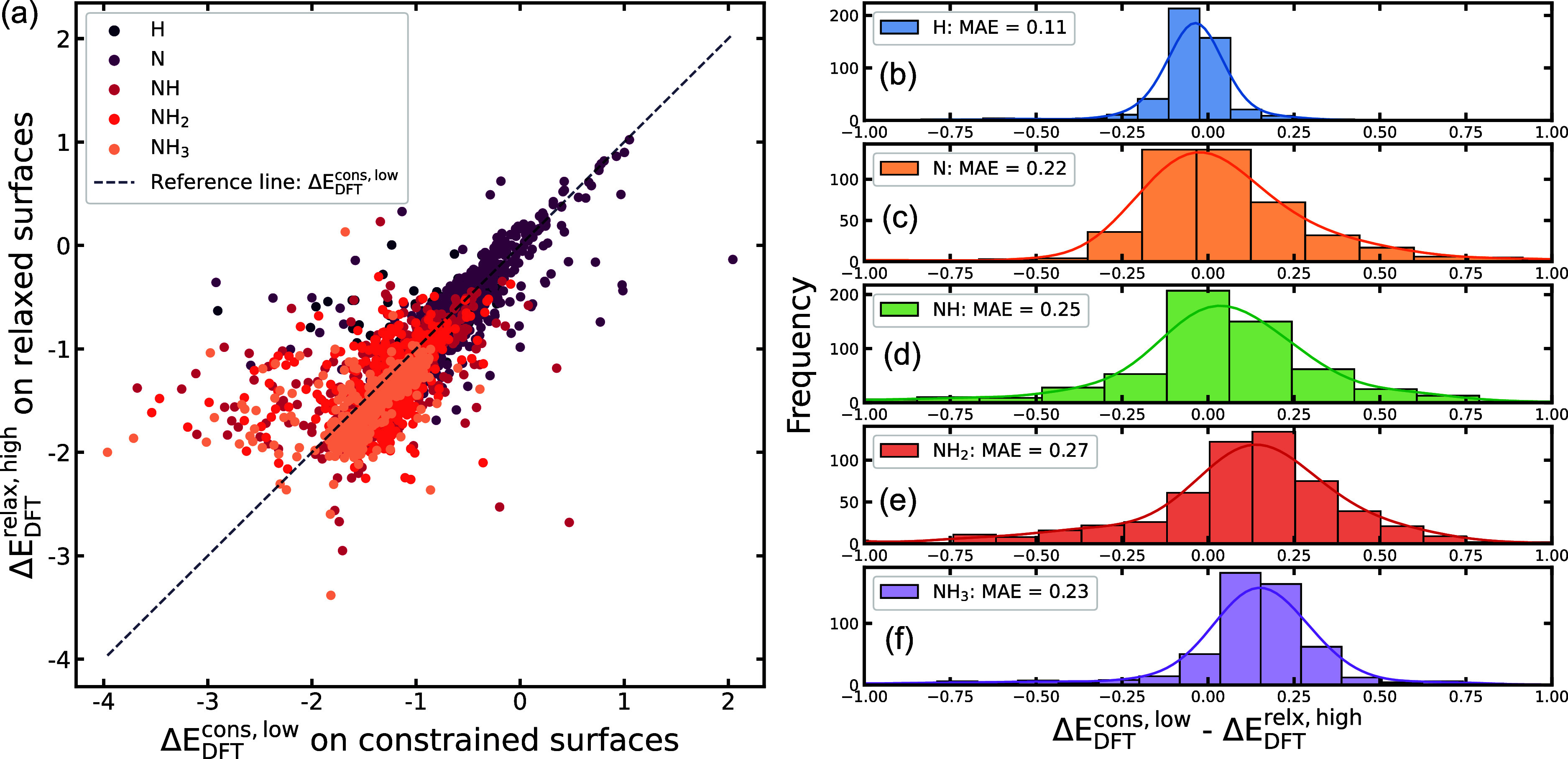
Comparison of Δ*E*_DFT_^cons,low^ vs Δ*E*_DFT_^relax,high^. (a) Parity plot of Δ*E*_DFT_^cons,low^ and Δ*E*_DFT_^relax,high^ for each pair of constrained and relaxed CoMoFeNiCu binding sites. Distribution of errors between Δ*E*_DFT_^cons,low^ and Δ*E*_DFT_^relax,high^ for (b) H, (c) N, (d) NH, (e) NH_2_, and (f) NH_3_ adsorbates outliers outside the range between −1 and 1 eV are not shown in the plot but were included into the MAE calculations.

As depicted in Figure S1, Δ*E*_DFT_^cons,low^ exhibits a wider range of values than the analogous Δ*E*_DFT_^relax,high^. Specifically, Δ*E*_DFT_^relax,high^ values vary from −1.2 to 0 eV for H, −2.0 to 1.0 eV for N, −3.0 to 0.2 eV for NH, −2.3 to −0.3 eV for NH_2_, and −3.5 to 0 eV for NH_3_. These distributions of Δ*E*_DFT_^relax,high^ highlight the diversity of the adsorption configurations in our database. Consistent with previous results, we reproduce the preferential binding of H, N, and NH species at the threefold sites, the bridge adsorption of NH_2_, and the on-top adsorption of NH_3_.^14^ For the NH_2_ species, depending on the local environment, we observe that all threefold sites (10%), on-top sites (7%), and bridge sites (83%) can be occupied. Although the computational cost of acquiring Δ*E*_DFT_^cons,low^ is 4.26 times cheaper than the Δ*E*_DFT_^relax,high^ calculations, Figure 3a shows that the Δ*E*_DFT_^cons,low^ predictions are only moderately correlated to the Δ*E*_DFT_^relax,high^ with an overall *R*^2^ value of 0.58. Specifically, Figure 3b–f shows the distribution of errors in two calculations (Δ*E*_DFT_^cons,low^ – Δ*E*_DFT_^relax,high^) for each adsorbate. We observe the relatively high MAEs consistently across all adsorbates, except for H, because of its narrower range of adsorption energy distribution. Despite this weak correlation, we now explore whether it is possible to develop ML models to bridge the accuracy gap between the two calculations. This is illustrated using the GLaSS descriptor below.

**Figure 4:**
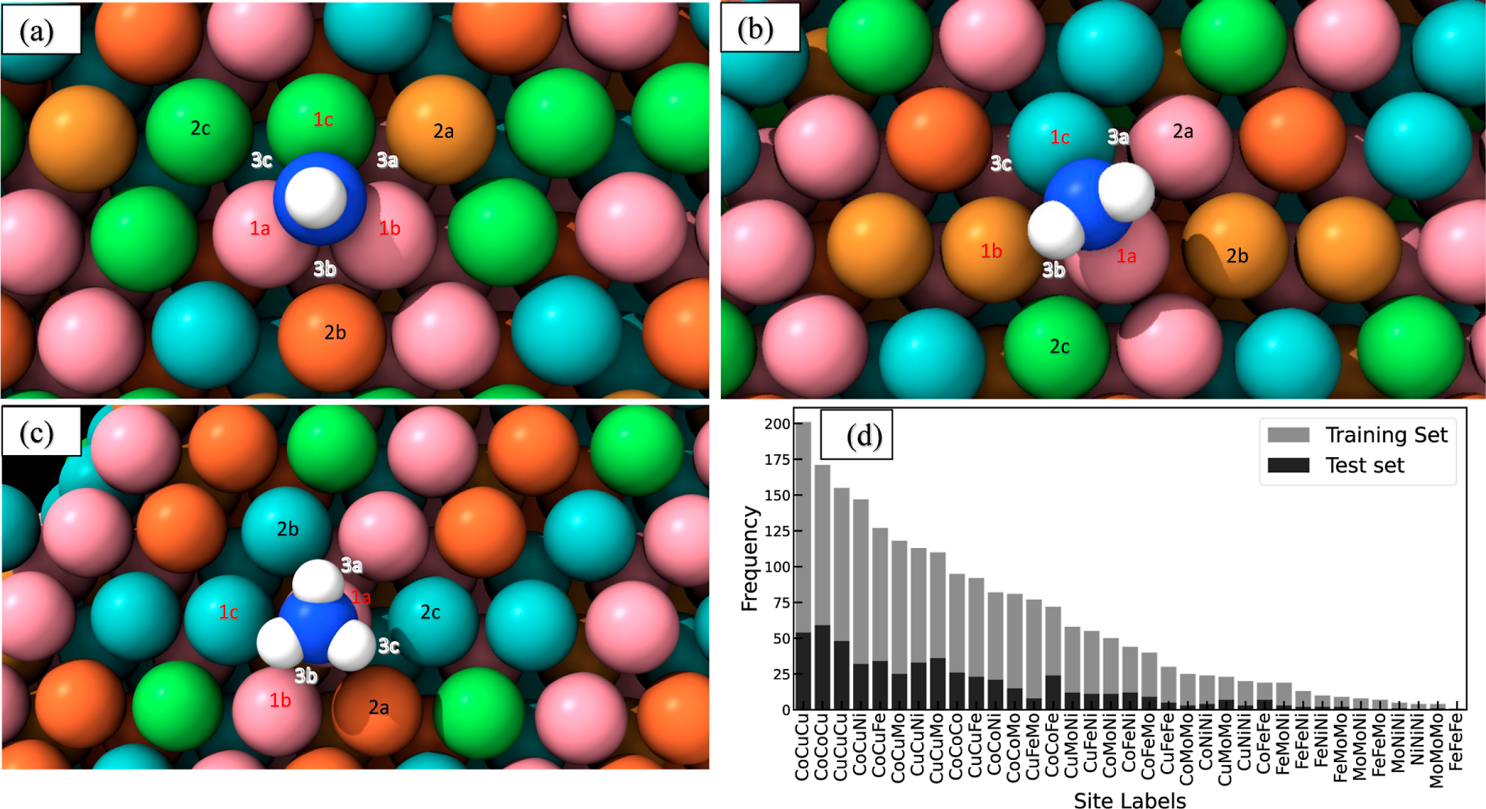
Illustration of the three possible site motifs with atoms considered in the GLaSS descriptor and the histogram distribution of binding site labels. (abc): The GLaSS applied to three types of binding sites: (a) hollow site, (b) bridge site, and (c) on-top site. The labels “1”, “2”, and “3” correspond to the closest three surface atoms, the next three closest surface atoms, and the nearest three subsurface atoms to the adsorbate, respectively. Color scheme of the atoms: Co atom: pink, Mo atom: light blue, Ni atom: green, Cu atom: brown, Fe atom: orange, N atom: deep blue, and H atom: white. (d) Histogram distribution of the binding site labels formed by the possible combinations of three zone-1 atoms in the training set and test set.

Figure 4a–c provides schematic representations of hollow, bridge, and on-top sites observed from the optimized CoMoFeNiCu binding surfaces. The GLaSS descriptor is determined by a fixed size of nine atoms and consists of a 37-dimension feature vector that captures the local environment of the adsorption site using atomic distances, angles, and dihedrals. First, the local atomic environment is divided into three zones based on the proximity of the HEA atoms to the adsorbate. Specifically, zone-1 represents the closest three atoms on the first layer of the surface (atoms labeled as “la”, “1b”, and “1c”), zone-2 represents the next three closest atoms within the same layer (“2a”, “2b”, and “2c”), and zone-3 refers to the closest three atoms in the subsurface layer (“3a”, “3b”, and “3c”). Importantly, the labeling “a”, “b”, and “c” within each zone signifies their membership and alphabetical ordering in GLaSS descriptor. We utilize a default alphabetical ordering based on the chemical composition of the HEA system, i.e., the features are sequentially ordered by Co, Cu, Fe, Mo, and Ni for the CoMoFeNiCu HEAs across each previously defined zone.

To illustrate this protocol, Table 2 shows the detailed implementation of the GLaSS descriptor for the bridge site, as depicted in Figure 4b. The first 27 features are associated with the local element-specific environment of the adsorbing atom with its three zone-1 neighbors: we use 15 distances, 9 angles, and 3 dihedral angles. The distances are represented by concatenating three one-hot-encoded feature vectors (the first, second, and third atom distance in Table 2); the length and ordering of the vector correspond to the number of distinct elements present in the CoMoFeNiCu HEA. The angle and dihedral terms account for all three-body and four-body interactions, respectively, comprising the adsorbing atom and the three zone-1 atoms. The zone-2 features are encoded as the sum-of-distances of the adsorbing atom from each of the zone-2 atoms; this results in five sum-of-distances corresponding to each element (i.e., zone-2 sum of distances for Co, Cu, Fe, Mo, and Ni in Table 2). An analogous approach for zone-3 provides the final five features of the GLaSS descriptor (zone-3 sum of distances in Table 2).

**Table 2:** Example with Detailed Geometric Meaning of Each Feature in the GLaSS Descriptor Applied to the Adsorption configuration of Figure 4b (NH_2_ Binding on the Bridge Site)^a^

		Co	Cu	Fe	Mo	Ni
zone-1	first atom distance	2.06 (A-1a)	0	0	0	0
	second atom distance	0	2.93 (A-1b)	0	0	0
	third atom distance	0	0	0	2.12 (A-1c)	0
	angle_0a1/a01/a10	angle_0a2/a02/a20	angle_1a2/a12/a21			
	73.7/63.9/42.4 (∠1a-A-1b/∠A-1a-1b/∠A-1b-1a)	51.9/78.3/49.7 (∠1a-A-1c/∠A-1a-1c/∠A-1c-1a)	43.6/64.2/72.2 (∠1b-A-1c/∠A-1b-1c/∠A-1c-1b)			
	dihedral_a012	dihedral_a021	dihedral_a120			
	55.1 (∠A-1a → ∠1b-1c)	90.8 (∠A-1a → ∠1c-1b)	53.3 (∠A-1a → ∠1b-1c)			

		Co	Cu	Fe	Mo	Ni
zone-2	sum of distances	HTML]FFFFFF	HTML]FFFFFF	HTML]FFFFFF	HTML]FFFFFF 0	HTML]FFFFFF

		Co	Cu	Fe	Mo	Ni
zone-3	sum of distances	HTML]FFFFFF	0	0	0	0

a Features are ordered by zones 1–3 and geometric properties. Symbol A refers the adsorbing atom (N).

The development of the GLaSS descriptor is inspired by the previous work of generalized coordination number (GCN)^22,39^ and the linearly parameterized representation of adsorption sites proposed by Batchelor et al.^11,30^ Here, we use a one-hot encoding philosophy, where the binary yes/no assignments are replaced by the distances (for zone-1) and sum-of-distances (for zone-2 and zone-3) to capture the differences in the adsorbate binding. Thus, while previous strategies require a priori labeling of the adsorbate binding site, the GLaSS encoding can be generalized more easily to any adsorption site. However, the critical shortcoming of this approach is that a DFT-optimized structure is required to obtain the geometric parameters that form the descriptor. Before outlining our strategy to overcome this challenge, we first demonstrate the efficacy of this descriptor for predicting the Δ*E*_DFT_^relax,high^ of the five adsorbates that are relevant for the NH_3_ decomposition reaction.

In addition to the aforementioned database comprising 2109 CoMoFeNiCu HEA sites that form the training set, an additional 534 (25.3% of the training set) HEA sites were considered as a test set. Figure 4d shows the distribution of binding site labels, derived from the combinations of three zone-1 atoms for each binding configuration, for both the training and test sets. We note that all 35 possible combinations of zone-1 atoms, considering the presence of five elements, were sampled and included in our database for training purposes. However, in the test set, 32 out of the 35 possible combinations were observed, with the exceptions of the “FeFeMo”, “FeFeFe”, and “NiNiNi” labels. These three cases are considered rare owing to the limited presence of Fe and Ni in the CoMoFeNiCu HEA composition. The frequency of these enumerated combinations varies, reflecting the composition-dependent distribution of the binding configurations within the HEA. Overall, the training and test sets exhibit a well-distributed diversity of local environments.

The GLaSS descriptor was used to encode the optimized relaxed binding configurations present within the training set (i.e., GLaSS^relax,high^). Subsequently, GLaSS^relax,high^ and their corresponding Δ*E*_DFT_^relax,high^ was used to develop a series of ML models using the Extreme Gradient Boosting algorithm (XGBoost).^40^ XGBoost uses an ensemble of parallel tree-boosting machines that enables the visualization of feature importance and cross-feature relationships. Hyperparameter tuning was implemented using Optuna,^41^ which is an automatic framework designed for optimizing hyperparameters in complex search spaces. Five separate XGBoost models were trained for each adsorbate type. To optimize the performance of each XGBoost model, a fivefold CV was performed within the training set. During this process, onefold of the data set was used as a validation set, enabling Optuna-enabled automatic hyperparameter tuning to achieve the best performance for each XGBoost model.

**Figure 5:**
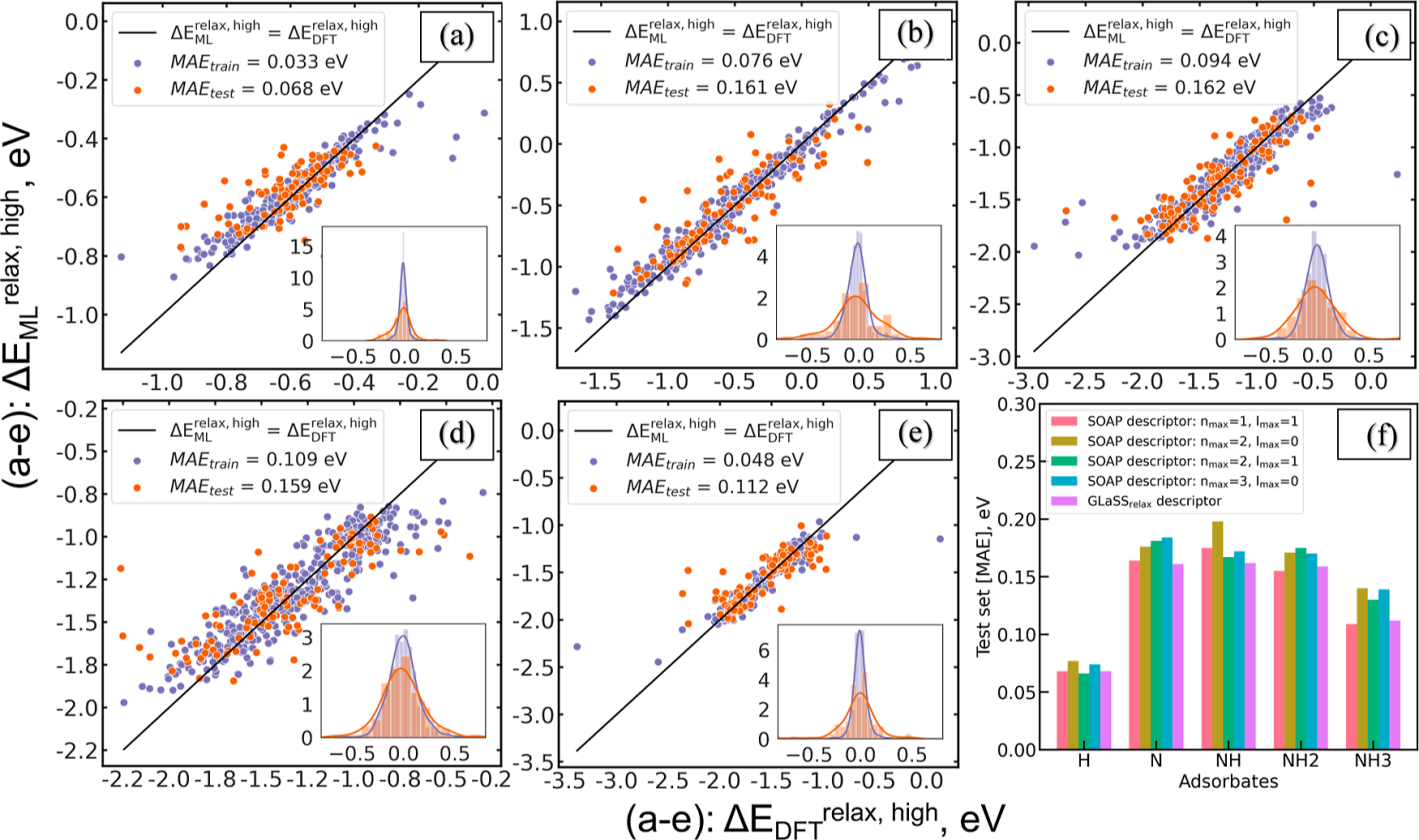
Performance evaluation of GLaSS^relax,high^ descriptors for each type of adsorbate (a–e): DFT-calculated vs XGBoost-predicted adsorption energies for each adsorbate. Each parity plot indicates (a) H, (b) N, (c) NH, (d) NH_2_, and (e) NH_3_. The purple dots refer the training set binding sites, and the orange dots refer the test set binding sites. The insets are the distribution of the prediction errors. (f) Comparison plot of model performance using MAEs on the same test set across four types of SOAP descriptors and our GLaSS^relax,high^ descriptor.

Figure 5a–e summarizes the efficacy of the GLaSS^relax,high^/XGBoost model in predicting the Δ*E*_DFT_^relax,high^ of the five adsorbates considered in this study. The XGBoost models achieved MAEs of 0.068 and 0.112 eV for H and NH_3_ test sets, respectively, while a slightly higher MAEs is observed for N, NH, and NH_2_ test sets. The normally distributed absolute prediction errors, provided as insets within each parity plot, indicate the absence of systematic bias in our predictions. Although prior work achieved higher prediction accuracy using more complex CNN models and hybrid ensemble of descriptors,^14^ we note that this approach is advantageous due to the simplicity of the GLaSS descriptor and interpretability of the XGBoost algorithm. Note that these MAEs are obtained from the test set, which is somewhat different than the training set. Although higher MAEs are observed, this sampling strategy allows us to assess the transferability of the model to previously unseen configurations (e.g., “NiNiNi”). We anticipate that the performance of our models can be further improved by using a larger training set.

To further access the performance of the GLaSS descriptor, we used the above train/test data set to develop additional XGBoost models for each adsorbate using SOAP descriptors,^42−45^ as implemented in the DScribe software package.^31^ In this study, the cutoff radius of the local region (r_cut_) was fixed at 6 Å. We observed that previous work, particularly that of Jäger et al.,^43^ employed larger radial and angular basis functions. However, it is worth noting that our study encompasses six to seven distinct chemical element types within our HEA systems. In contrast, the prior study primarily focused on relatively simpler systems such as molybdenum disulfide and copper–gold clusters. As the SOAP features scale exponentially with the number of distinct elements in the system, the range of radial basis functions (*n*_max_) was set at 1–3, and the maximum degree of spherical harmonics (*l*_max_) was set to 0 or 1 (by imposing a soft constraint of *n*_max_ ≥ _max_ + 1) in this work. Consequently, we systematically selected *n*_max_/*l*_max_ combinations of 1/1, 2/0, 2/1, and 3/0, resulting in SOAP descriptor feature sizes ranging from 42 to 171 for H@HEA and N@HEA systems and from 56 to 231 for NH, NH_2_, and NH_3_ binding on HEA systems. We have compiled and provided parity plots for all of these models in Figures S2–S5. The comparison of model performance and the corresponding MAEs are summarized in Figure 5f. Encouragingly, the GLaSS^relax,high^ descriptor consistently outperformed all the SOAP descriptors with different combinations of *n*_max_ and *l*_max_ parameters, especially for systems consisting of N as binding species. However, we did observe that other geometric descriptors, like the Localized version of Many Body Tensor Representation,^46^ as briefly analyzed in the Supporting Information (Figure S6), may show better performance than our GLaSS^relax,high^ descriptor, especially with careful fine-tuning.

Given the potential of the GLaSS descriptor for achieving robust and reliable predictions for Δ*E*_DFT_^relax,high^, we further propose to take advantage of the already available low-accuracy DFT calculations, i.e., using an ensemble descriptor of GLaSS^cons,low^ and Δ*E*_DFT_^cons,low^ to predict the high-accuracy adsorption energies (i.e., Δ*E*_DFT_^relax,high^). This approach draws inspiration from the concept of scaling relations and aims to circumvent the computationally expensive geometric optimizations, thus reducing the overall computational costs associated with high-throughput surface screening. However, a significant constraint of the proposed approach lies in the fact that the ensemble descriptor still necessitates DFT calculations, thereby failing to achieve the desirable DFT-free attribute. To overcome this bottleneck, we trained a ML-FF using NEP^37^ interatomic potential with data from the SPEs during the calculation of Δ*E*_DFT_^cons,low^. We adopted the state-of-the-art NEP version 4 potential, which is made available in GPUMD package with superior performance regarding multicomponent atomistic systems.^38^

**Figure 6:**
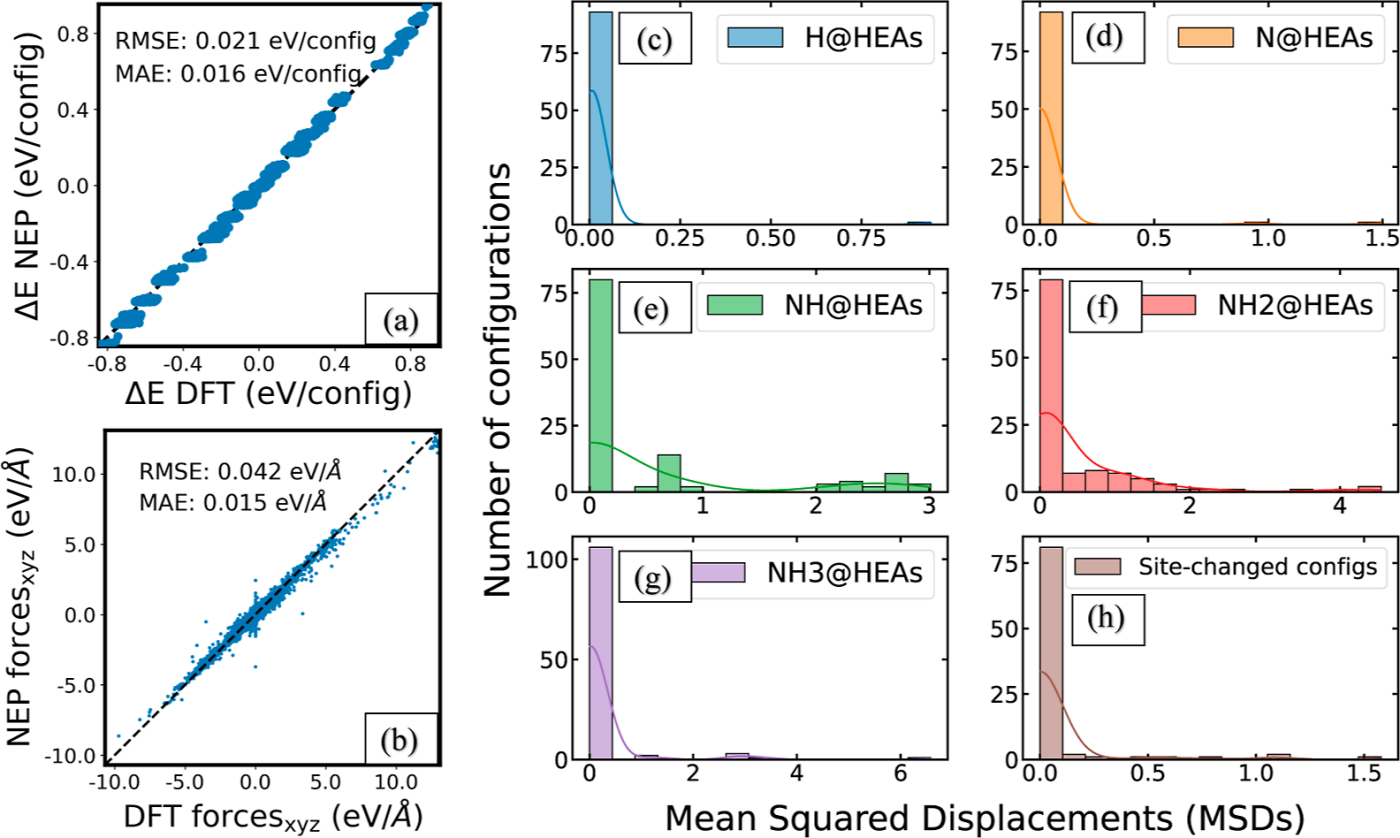
Overall performance assessment of NEP-ML-FF: the inherent model accuracy and its predictability of the optimized binding site configurations for the test set. (a,b) Parity plots comparing NEP-ML-FF predicted (a) configuration potential energies and (b) forces with corresponding DFT values. (c–g) Distribution of MSDs between the NEP-ML-FF-predicted sites and the DFT-optimized sites for each adsorbate. (h) Distribution of MSDs of the DFT-disagreed NEP-ML-FF-optimized binding configurations. MSDs were calculated between the original DFT-optimized configurations and their predicted sites after rerun by NEP-ML-FF.

The ML-FF aims to predict the energetically minimized binding configuration of the low-accuracy constrained surfaces. As discussed previously, a downsampling strategy was used to obtain 67,577 configurations. We adopted 90–10% train-test set split to train and validate the NEP-ML-FF. The parity plots comparing NEP predictions to DFT values for the potential energies (per binding configuration) and forces are shown in Figure 6a,b, respectively. NEP-ML-FF was found to be able to predict DFT values with excellent accuracy, as seen by the MAE of 1.6 × 10^–3^ eV/configuration for energies and 1.5 × 10^–3^ eV/Å for forces.

The validated NEP-ML-FF is subsequently applied for the optimization of the 534 initial configurations within the test set. The optimization process is facilitated by the FIRE local atomic structure optimization algorithm,^47^ which is implemented in the Atomic Simulation Environment (ASE) software package.^48^ We then computed the mean squared displacements (MSDs) between the sites predicted by the NEP-ML-FF and the sites optimized through DFT calculations. Details of the MSDs calculations are provided in the Methods section. The distribution of MSDs for each adsorbate is presented in Figure 6c–g. A visual comparison between a pair of representative binding site geometries, each optimized separately using DFT and NEP-ML-FF, is presented in Figure S7. Notably, the NEP-ML-FF model effectively reproduces DFT-optimized site geometries, evident from the fact that approximately 83% of the binding sites within the test set exhibit MSD values below 0.2. This result underscores a significant degree of structural similarity. Among the five adsorbates, it is noteworthy that NEP-ML-FF faces challenges in replicating DFT results for NH and NH_2_. For these two adsorbates, 37 out of 117 NEP-ML-FF-predicted sites and 42 out of 116 such sites, respectively, exhibit MSD values greater than the predefined threshold of 0.2, indicative of disagreements with DFT results.

While the observation of site changes during independent trials of geometry optimizations might not come as a surprise, owing to the presence of multiple locally optimal sites for the HEA surfaces, we took an additional step to improve the robustness and effectiveness of the NEP-ML-FF. Specifically, we employed the DFT-optimized configurations as inputs for a rerun of the NEP-ML-FF. The resulting distribution of the MSDs, calculated before and after the NEP-ML-FF, is depicted in Figure 6h. Among the 90 binding sites where prior site changes were observed, a remarkable 83 sites displayed MSD values below 0.2, alongside the corresponding optimization steps spanning from 2 to 10. In essence, our NEP-ML-FF demonstrated 92% success in reproducing the DFT-predicted binding site, which suggests that this approach can be used to create the geometry-based GLaSS descriptor to predict Δ*E*_DFT_^relax,high^.

While the DFT-free GLaSS descriptor (denoted as GLaSS_NEP_^cons,low^) can be directly obtained from the NEP-ML-FF, the energetic component of the ensemble descriptor (denoted as Δ*E*_ML_^cons,low^) necessitates prediction through a separate XGBoost model, which is trained using the DFT-optimized GLaSS^cons,low^ and Δ*E*_DFT_^cons,low^. In line with our aim for a completely DFT-free approach, the inputs for the XGBoost model should exclusively originate from the NEP-ML-FF. While we constructed the test set using paired data of GLaSS_NEP_^cons,low^ against Δ*E*_DFT_^cons,low^ for all 534 sites in our test set, we note that the site changes discussed above may worsen the prediction errors. Specifically, the MAE of the XGBoost model for predicting Δ*E*_ML_^cons,low^ was 0.230 eV, exclusively based on the binding sites that did not undergo site changes (total of 444 points). The parity plot is shown in Figure S8. We observe that this larger deviation is predominantly attributable to the presence of “rare” configurations, as evidenced by outliers in the distribution of the ΔE_DFT_^cons,low^ data set.

**Figure 7:**
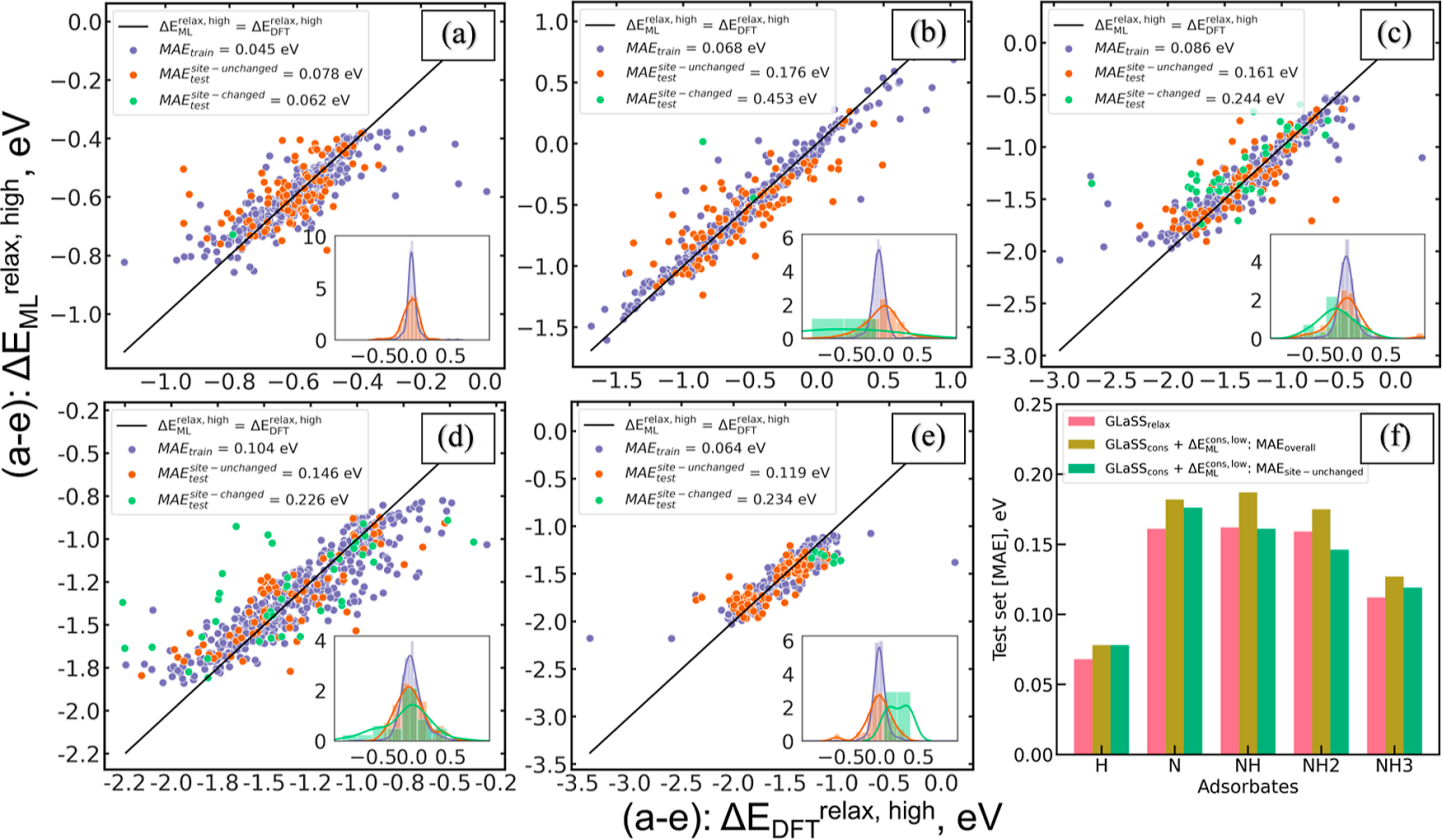
Performance evaluation of the GLaSS_NEP_^cons,low^ + Δ*E*_ML_^cons,low^ ensemble descriptor in the prediction of Δ*E*_DFT_^relax,high^ for each type of adsorbate decomposed by site changed and unchanged scenarios and comparison with models solely using the GLaSS^relax,high^ descriptor. (a–e) DFT-calculated vs XGBoost-predicted adsorption energies for each adsorbate. Each parity plot indicates (a) H, (b) N, (c) NH, (d) NH_2_, and (e) NH_3_. The purple dots refer to the training set binding sites, and the green/orange dots refer to the test set binding sites with/without site changes, respectively. (f) Comparison plot of model performance using MAEs for individual adsorbate. Yellow bins: overall MAEs achieved by the GLaSS_NEP_^cons,low^ + ΔEMLcons,low ensemble descriptor; green bins: decomposed overall MAEs for site-unchanged binding sites; red bins: reference of MAEs achieved by solely using the GLaSS^relax,high^ descriptor.

Figure 7a–e summarizes the effectiveness of the ensemble descriptor/XGBoost model in predicting high-accuracy adsorption energies on a relaxed slab for the same five test sets of adsorbates. In comparison to previous models that exclusively utilized the GLaSS^relax,high^ descriptor to encode optimized relaxed binding configurations, the overall MAEs when employing the ensemble descriptor tend to be systematically higher, with the smallest and largest MAE deviations of 10 and 25 meV for the H and NH models, respectively. However, the overall MAEs still fall within the acceptable threshold of 0.2 eV for reproducing DFT-level accuracy. To address the issues with the site changes, we further decompose the overall MAEs based on binding sites with and without site changes. For the 444 sites without site changes (represented by orange dots), similar MAEs with normally distributed absolute prediction errors were observed, comparable to the results obtained using the previous GLaSS^relax,high^ descriptor, as illustrated in Figure 7f. However, there is a noticeable bias in the perturbation of errors, with larger discrepancies to the Δ*E*_DFT_^relax,high^ for the 90 site-changed binding sites (represented by green dots in Figure 7a–e). These site-changed sites contributed significantly to and explained the overall higher MAEs when employing the ensemble descriptor. Nevertheless, considering the benefits gained from the overall DFT-free approach, the utilization of the GLaSS_NEP_^cons,low^ + Δ*E*_ML_^cons,low^ ensemble descriptor for predicting previously unobserved binding sites within HEAs remains a favorable strategy.

**Figure 8:**
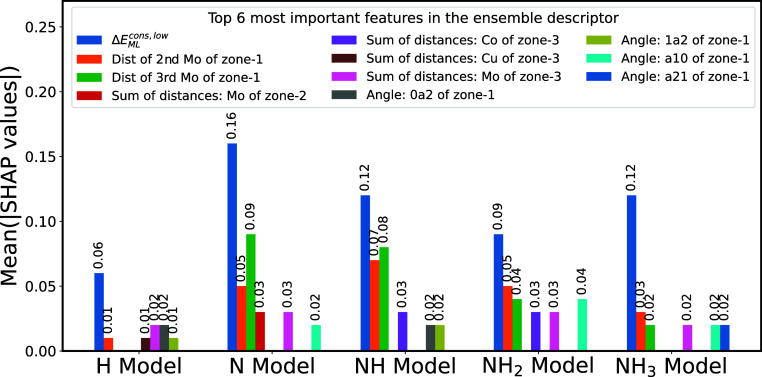
Ensemble bar plot of the top six most important features and their Shapley values in the GLaSS_NEP_^cons,low^ + ΔE_ML_^cons,low^ ensemble descriptor, calculated and ranked based on the TreeSHAP method.

Since XGBoost is a specific implementation of the gradient boosted trees algorithm, we employed the Tree-SHapley Additive exPlanations (TreeSHAP) method,^49,50^ tailored for tree-based ML models, to evaluate the feature importance of encoded optimized constrained binding configurations (GLaSS_NEP_^cons,low^) + Δ*E*_ML_^cons,low^ ensemble descriptor. To obtain a comprehensive assessment of feature importance, we calculated the average absolute Shapley values for each feature across the test set and organized the features in descending order of importance. We then visualized the top six most influential features in an ensemble bar plot for all five adsorbates, as illustrated in Figure 8. From the figure, it is evident that the energetic descriptor Δ*E*_ML_^cons,low^ displayed the highest Shapley values among the five models and significantly exceeded the second most important feature, which belonged to the GLaSS descriptor. Among the features within the GLaSS descriptor, the following features were identified as the most influential in explaining the predictability of Δ*E*_DFT_^relax,high^: distances of zone-1 Mo atoms to the adsorbing atom, four types of angles within zone-1, sum of distances of zone-2 Mo atoms, and sum-of-distances features for three zone-3 atoms (Co, Cu, and Mo). This overarching Shapley analysis underscores the substantial contribution of the energetic descriptor Δ*E*_ML_^cons,low^, in addition to geometric features, when predicting our target Δ*E*_DFT_^relax,high^.

## Conclusions

This work aims to address the challenge of developing interpretable ML-based models when access to large-scale computational resources is limited. Specifically, we have presented a cost-effective workflow that synergistically combines interpretable ML models (e.g., XGBoost) and ML-FFs (e.g., NEP) to predict high-accuracy adsorption energies for CoMoFeNiCu HEA catalysts using a daisy-chained approach. This is made possible by using three specific modifications to typical DFT workflows used within the field. First, we use a sequential multistep optimization protocol to reduce the computational cost of generating large DFT data sets. Second, we introduce a new descriptor (called GLaSS) that can be generalized to arbitrary surface binding sites. Third, we use low-quality DFT binding energies as an energetic descriptor in the XGBoost model. More importantly, these low-cost DFT optimization trajectories are also repurposed to develop a ML-FF that provides the geometric information necessary for creating the GLaSS descriptor. Taken together, this study illustrates how cheap DFT calculations and appropriately designed descriptors can be used to develop useful models for predicting high-quality adsorption energies at significantly lower computational costs. Although this work has focused on developing a cheap predictive model for one specific HEA composition (i.e., CoMoFeNiCu), we anticipate that our resource-efficient training philosophy may be broadly relevant to the larger surface catalysis community.

## Supporting Information Available

Histograms of the distribution of DFT-calculated adsorption energies for five adsorbates, parity plots of predicting adsorption energies using several other models, additional plots of model performance, comparison of the binding configurations, and training loss curves for the XGBoost models (PDF)
