# Supplementary material for: Developing Cheap but Useful Machine Learning-Based Models for Investigating High-Entropy Alloy Catalysts
Source: Langmuir. 2024 Feb 5;40(7):3691–701. doi: 10.1021/acs.langmuir.3c03401 (PMC10883032; doi:10.1021/acs.langmuir.3c03401)
Supplement: Supplementary file 1 — la3c03401_si_001.pdf [file la3c03401_si_001.pdf]

Developing Cheap but Useful Machine Learning based Models for Investigating High-Entropy Alloy Catalysts

Chenghan Sun, Rajat Goel, and Ambarish R Kulkarni*

Department of Chemical Engineering, University of California, Davis

E-mail: arkulkarni@ucdavis.edu

Supporting Information Available

Distribution of DFT calculated adsorption energies for all adsorbates shown in Fig: S1. $\Delta E_{DFT}^{cons,low}$ exhibits wider range of values than $\Delta E_{DFT}^{relax,high}$

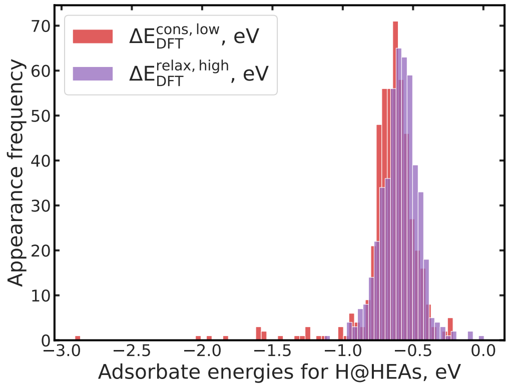

(a)

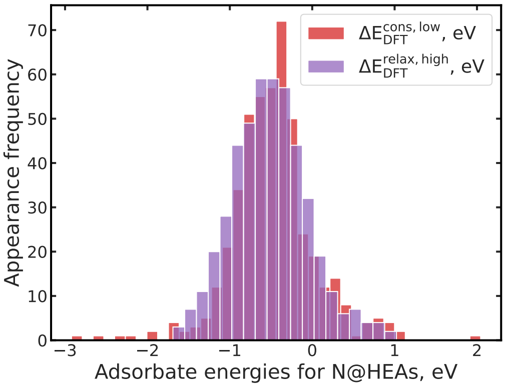

(b)

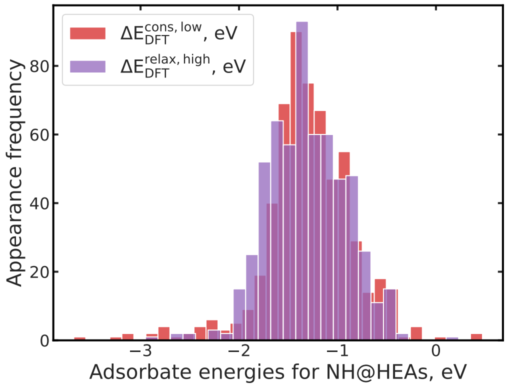

(c)

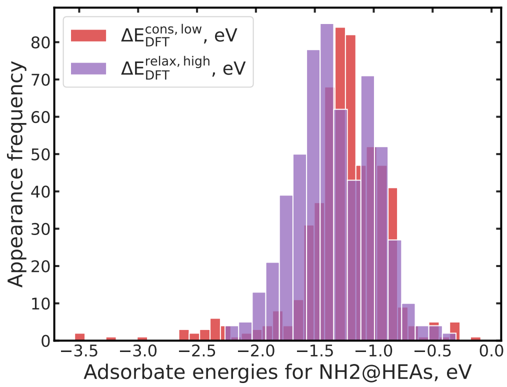

(d)

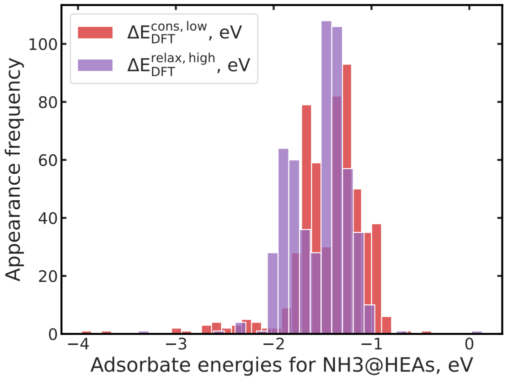

(e)

Figure (S1) **Histograms of the distribution of DFT-calculated adsorption energies for five adsorbates.** Specifically. (a): H, (b): N, (c): NH, (d): NH_2, (e): NH_3 on both constrained and relaxed CoMoFeNiCu HEA surfaces.

Comparison of DFT adsorption energies to SOAP descriptors (n_{max}=1, l_{max}=1) (Fig: S2), (n_{max}=2, l_{max}=0) model (Fig: S3), (n_{max}=2, l_{max}=1) model (Fig: S4), (n_{max}=3, l_{max}=0) model (Fig: S5) model.

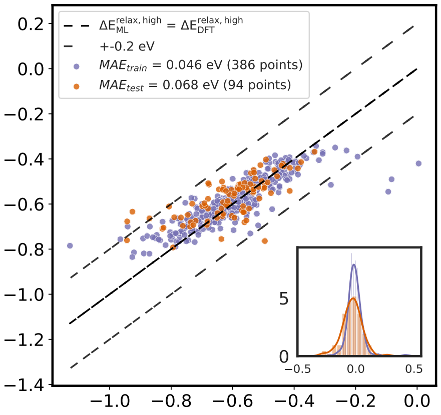

(a)

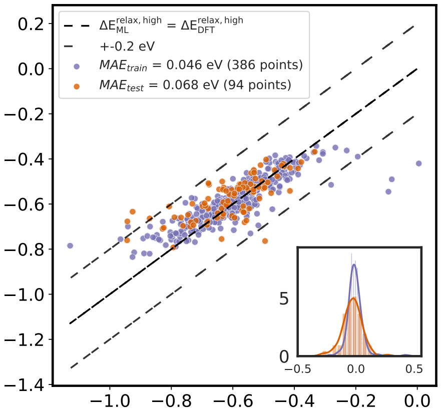

(b)

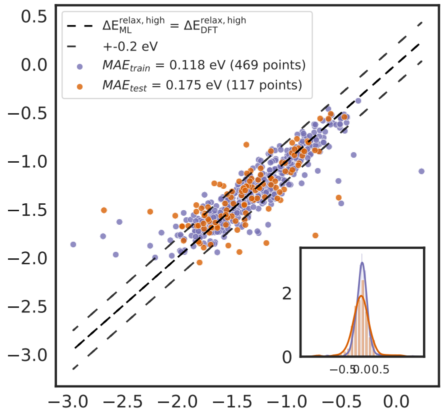

(c)

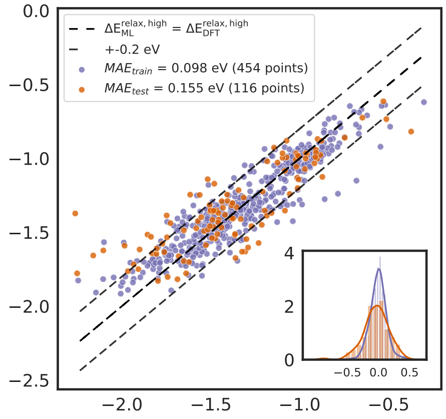

(d)

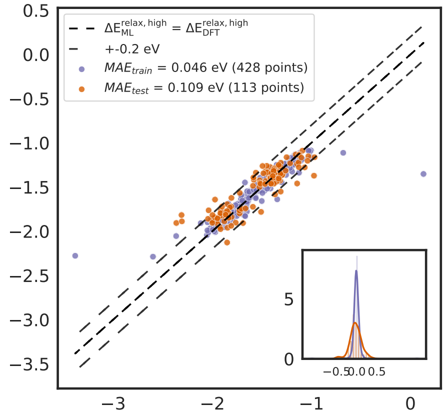

(e)

Figure (S2) **Parity plots of predicting adsorption energies using SOAP 11 (n_{max}=1, l_{max}=1) descriptors for relaxed HEA surfaces.** Specifically, (a) H (b) N (c) NH (d) NH_2(e) NH_3.

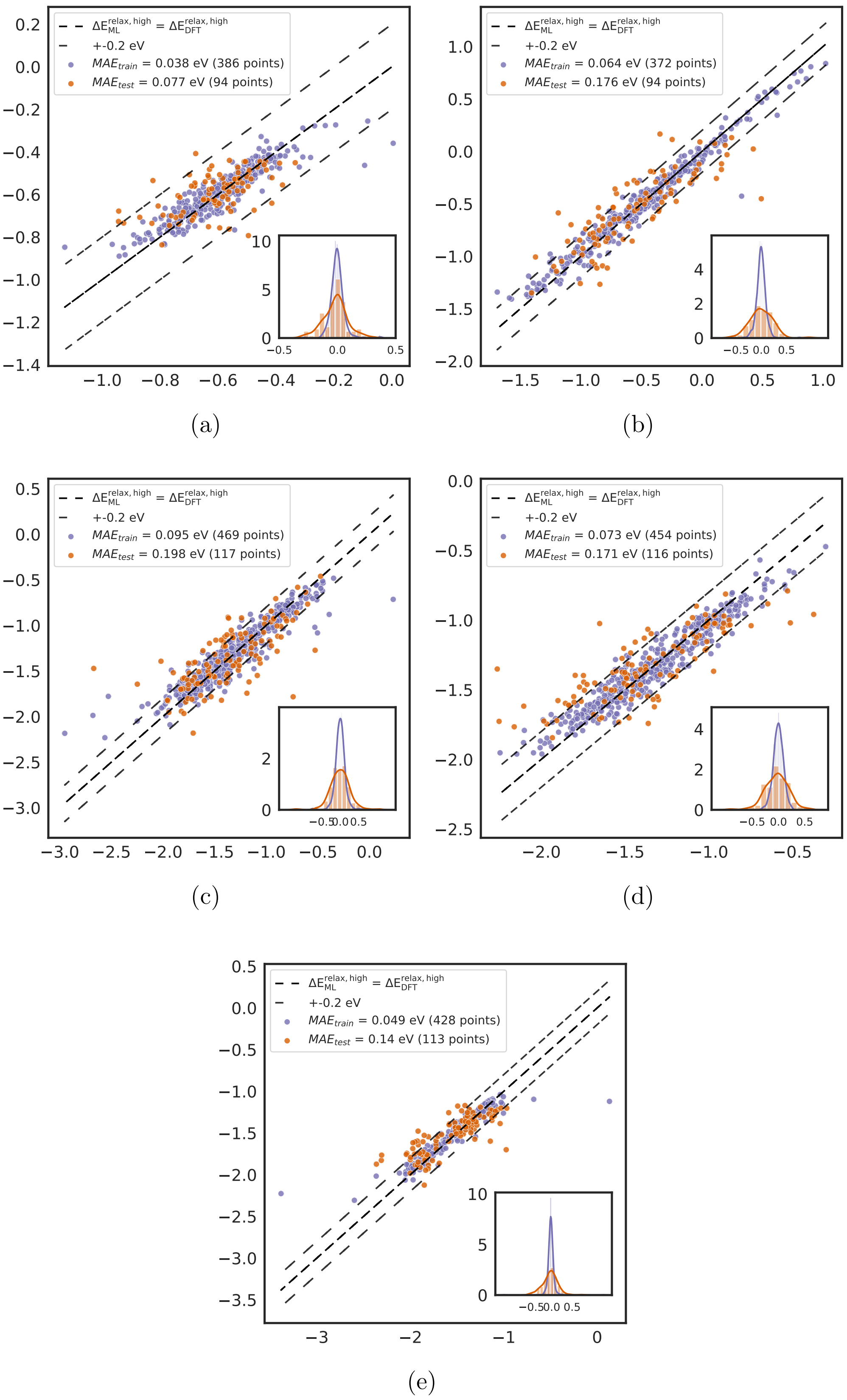

Figure (S3) **Parity plots of predicting adsorption energies using SOAP 20 (n_{max}=2, l_{max}=0) descriptors for relaxed HEA surfaces.** Specifically, (a) H (b) N (c) NH (d) NH_2(e) NH_3.

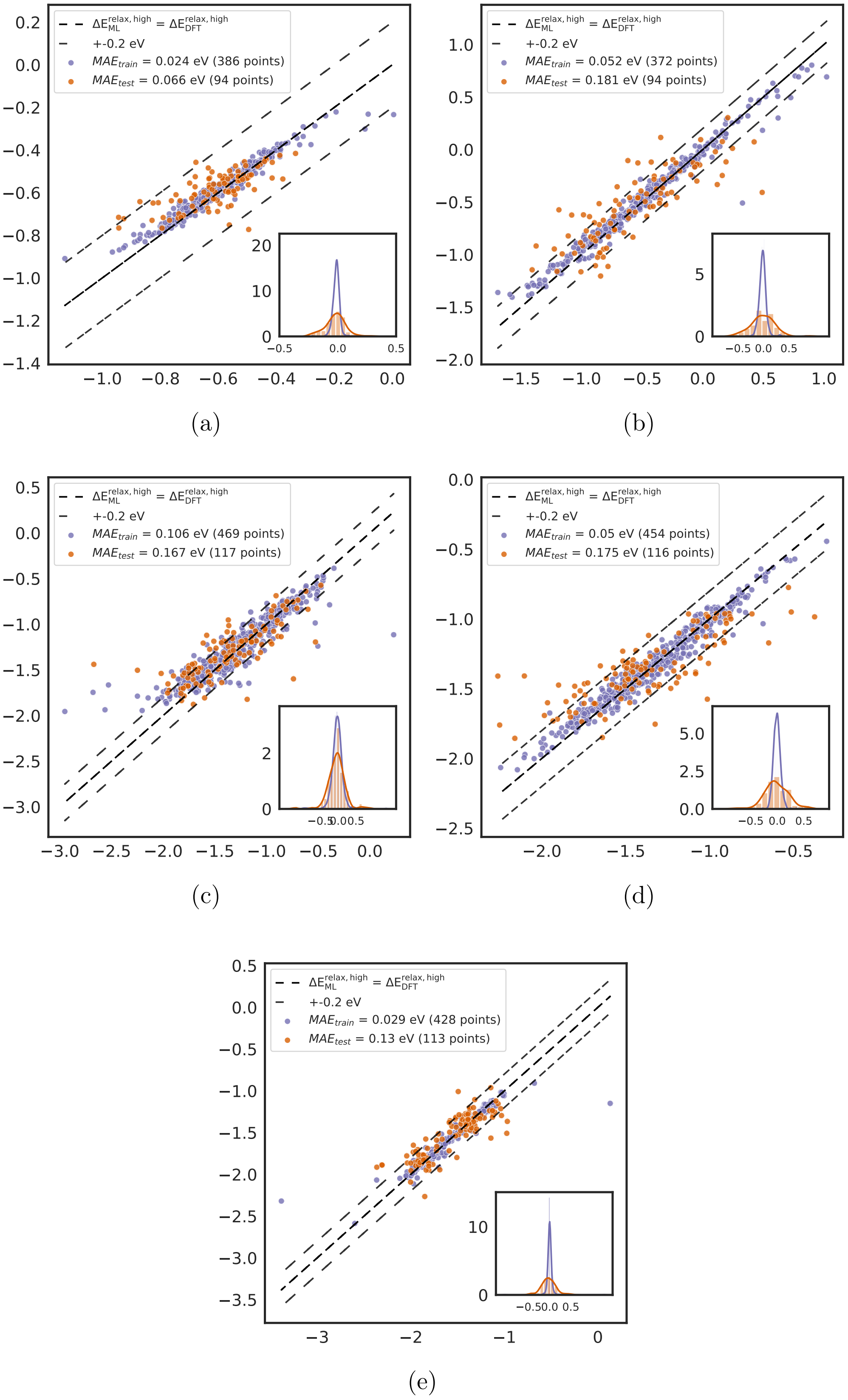

Figure (S4) **Parity plots of predicting adsorption energies using SOAP 21 (n_{max}=2, l_{max}=1) descriptors for relaxed HEA surfaces.** Specifically, (a) H (b) N (c) NH (d) NH_2(e) NH_3.

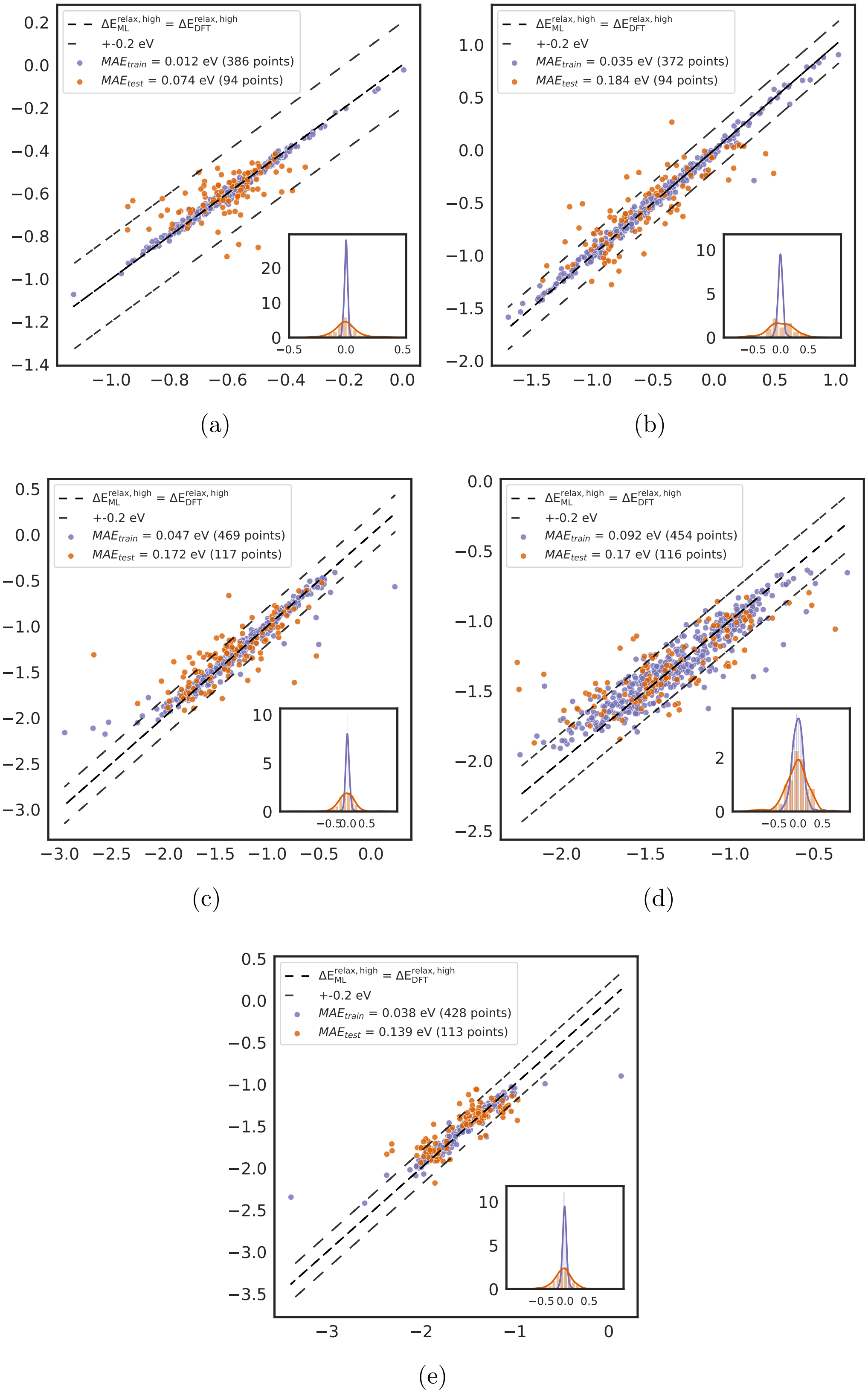

Figure (S5) **Parity plots of predicting adsorption energies using SOAP 30 (n_{max}=3, l_{max}=0) descriptors for relaxed HEA surfaces.** Specifically, (a) H (b) N (c) NH (d) NH_2(e) NH_3.

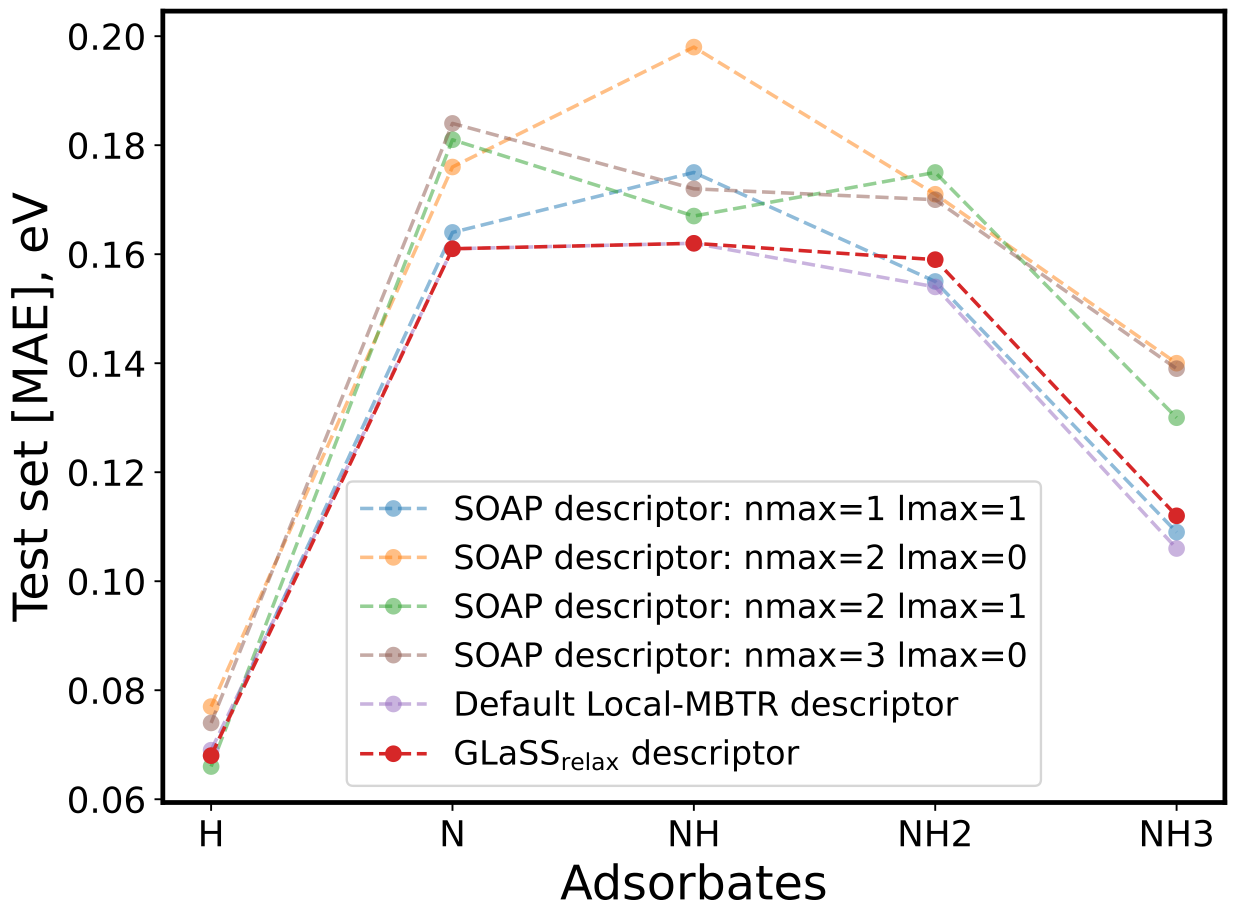

Figure (S6) **Additional comparison plot of model performance using MAEs on the same test set across four types of SOAP descriptors, the Local-MBTR descriptor, and our GLaSS^{relax,high} descriptor.**

In Fig: S6 assessment of different descriptors applied to our dataset, we employed the default configuration of the Local Many Body Tensor Representation (LMBTR) from the DScribe package. For the LMBTR descriptor, we employed a Gaussian distribution with a standard deviation of 0.1 to sample 30 points within a grid spanning distances from 1 to 6 Å. We used a scaling factor of 0.5 and a threshold of 0.01 for the weighting function. Our comparative analysis revealed that, for the H, N, and NH test sets, both the LMBTR and GLaSS^{relax,high} descriptors exhibited similar MAEs. However, it is noteworthy that the LMBTR descriptor achieved the lowest MAEs for the NH_2 and NH_3 test sets, recording values of 0.154 eV and 0.106 eV, respectively. In contrast, the GLaSS^{relax,high} descriptor yielded slightly higher MAEs of 0.159 eV and 0.112 eV for the same test sets.

It's worth mentioning that the LMBTR descriptor was not fine-tuned for this performance comparison. We anticipate that further optimization of the LMBTR descriptor, including adjustments to Gaussian parameters and increasing the number of samples within the grid, could potentially lead to improved performance on our dataset. However, it should be noted that constructing the LMBTR descriptor incurred higher computational cost and required more than 5 times the wall time of generating the SOAP descriptors in our study. As such, our GLaSS descriptor retains its advantages, offering geometric interpretability and a minimal computational burden for generating descriptors that provide a unified representation of diverse surface sites.

Fig: S7 shows the final binding configurations for 2 adosrbates using DFT and NEP-MLFF.

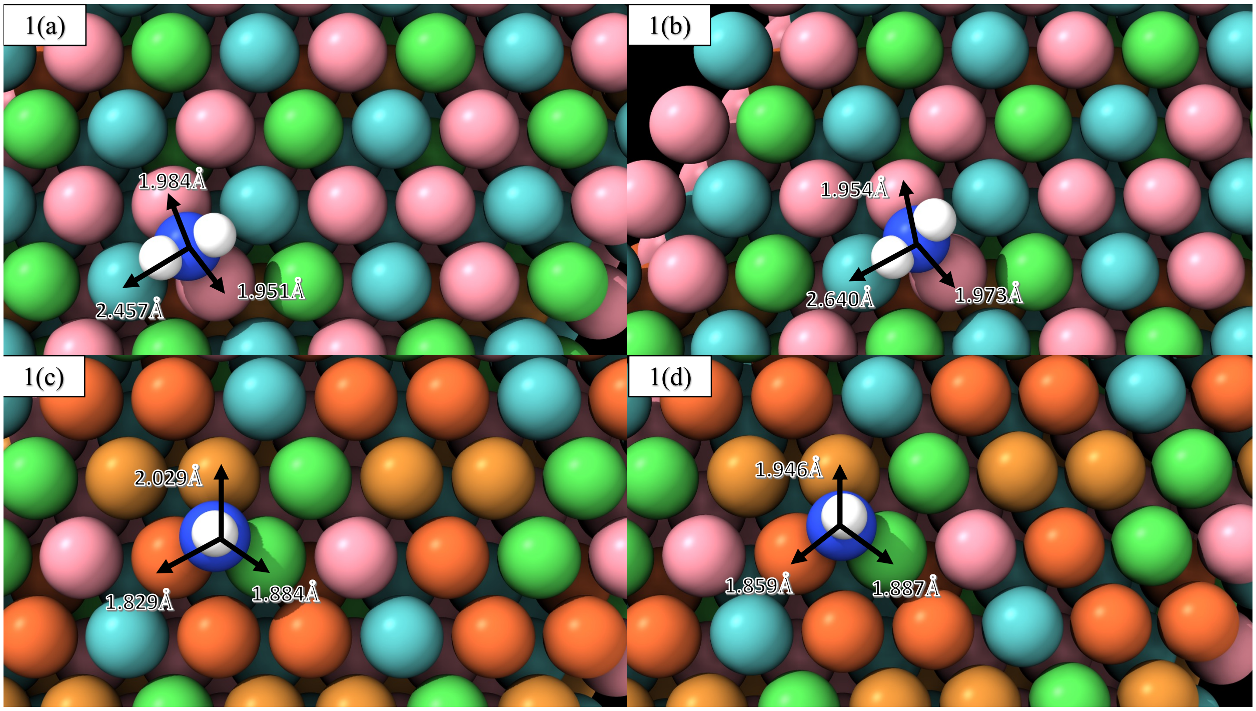

Figure (S7) **Comparison of the exemplified binding configurations after the DFT optimization and NEP-MLFF optimization with respect to the same initial structure.** (a-b): NH_2 binding on the $Co_{35}Mo_{35}Fe_{10}Ni_{10}Cu_{10}$ HEA surfaces (index: 3264 in the database). The configuration (a) is optimized by DFT, and the configuration (b) is optimized by NEP-MLFF; (c-d): NH binding on the $Co_{35}Mo_{35}Fe_{10}Ni_{10}Cu_{10}$ HEA surfaces (index: 3272 in the database). The configuration (a) is optimized by DFT, and the configuration (b) is optimized by NEP-MLFF. The distance between the adsorbing atom N to the nearest three surface atoms (zone-1) are highlighted.

Isolated $\Delta E_{ML}^{cons,low}$ for binding sites that did not undergo site changes in Fig: S8.

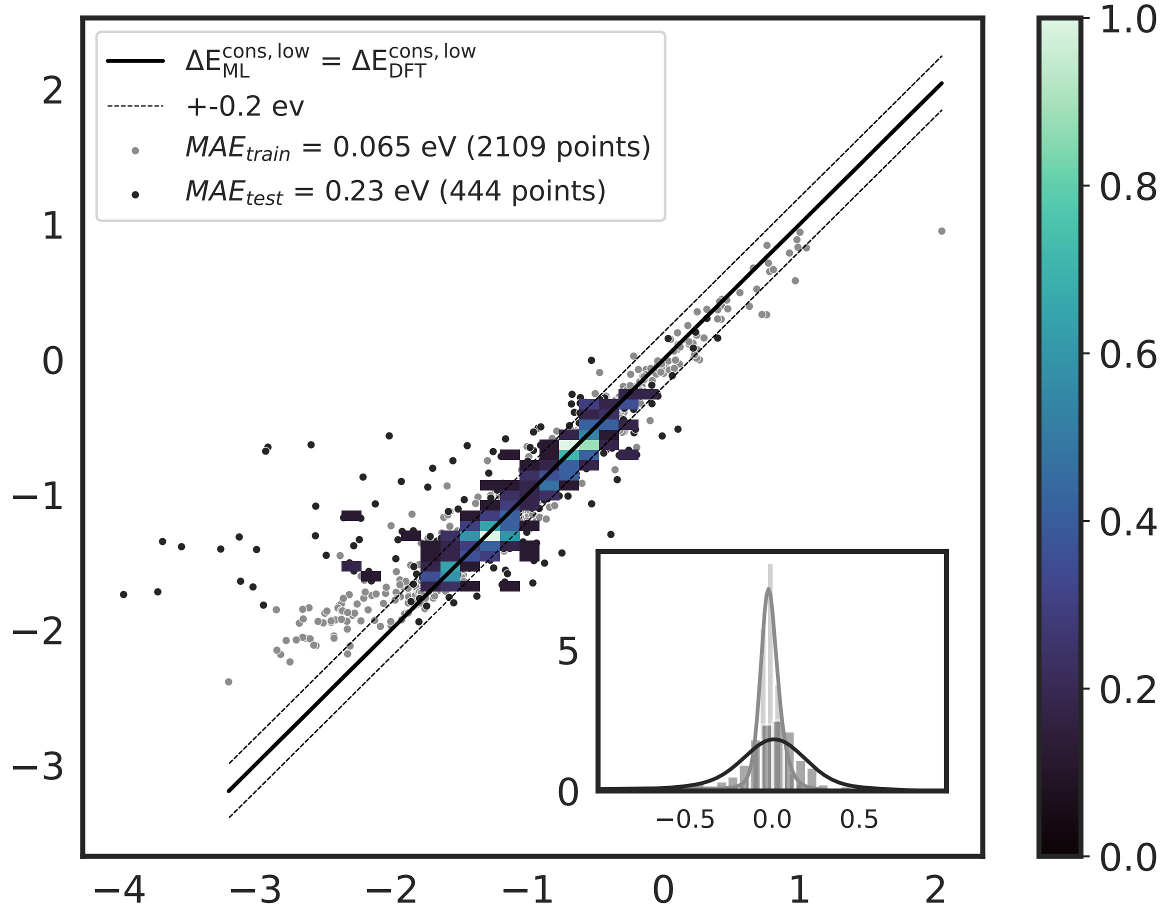

Figure (S8) **Parity plot illustrating the performance of the XGBoost model trained on pairs consisting of DFT-optimized GLaSS$^{\mathbf{cons,low}}$ and $\Delta \mathbf{E}_{\mathbf{DFT}}^{\mathbf{cons,low}}$.** The test set comprises paired data of GLaSS$_{NEP}^{cons,low}$ versus $\Delta E_{DFT}^{cons,low}$ for 444 out of the total 534 binding sites in the test set, specifically those without observed site changes. A side color bar which refers the data density is provided.

Training loss curves using GLaSS^{relax,high} descriptor (Fig: S9) and GLaSS$_{NEP}^{cons,low}$ + $\Delta E_{ML}^{cons,low}$ ensemble descriptor(Fig: S9) to predict $\Delta E_{DFT}^{relax,high}$.

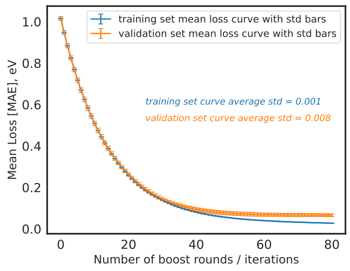

(a)

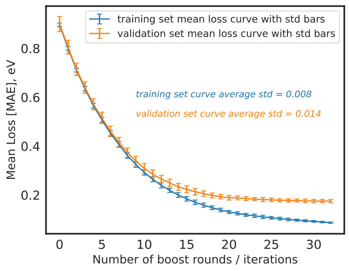

(b)

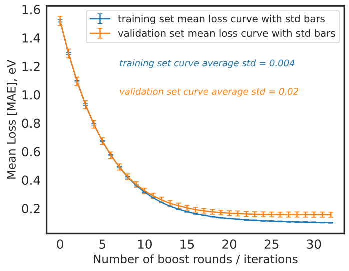

(c)

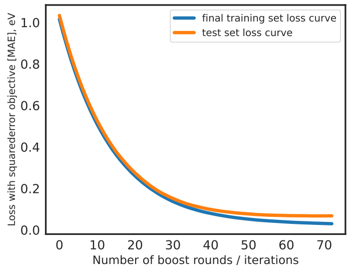

(d)

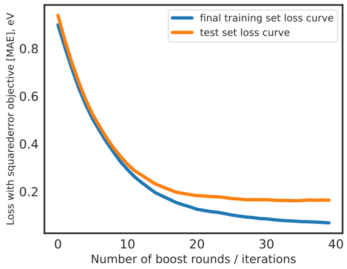

(e)

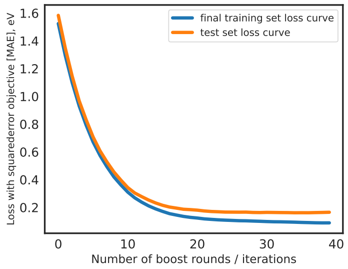

(f)

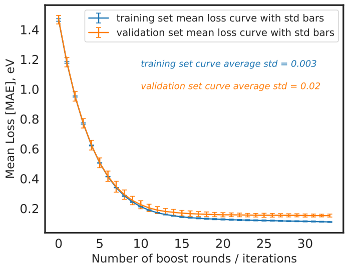

(g)

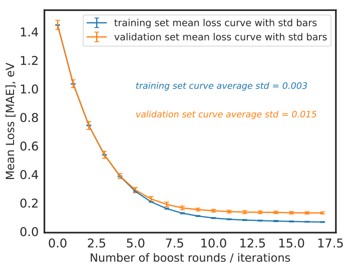

(h)

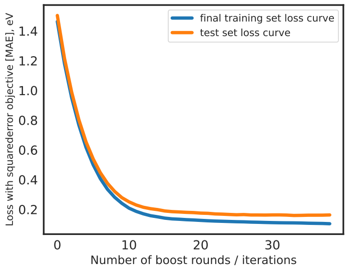

(i)

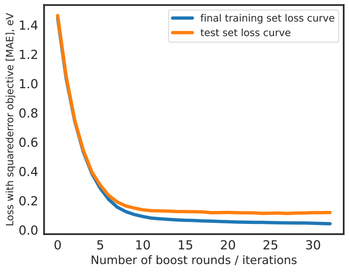

(j)

Figure (S9) **The training loss curves for the XGBoost models using the GLaSS$^{\mathbf{relax,high}}$ descriptor to predict $\Delta E_{DFT}^{relax,high}$.** Two types of loss curves are provided for each adsorbate. The first curve corresponds to the 5-fold cross-validation (CV) internally using the training data, and the error bar reflects the standard deviation across the 5-fold CV. The second curve represents the final model trained using all the training data and evaluated on the test set. The figure indices for each adsorbate type are as follows: H (a)&(d), N (b)&(e), NH (c)&(f), NH_2(g)&(i), NH_3 (h)&(j).

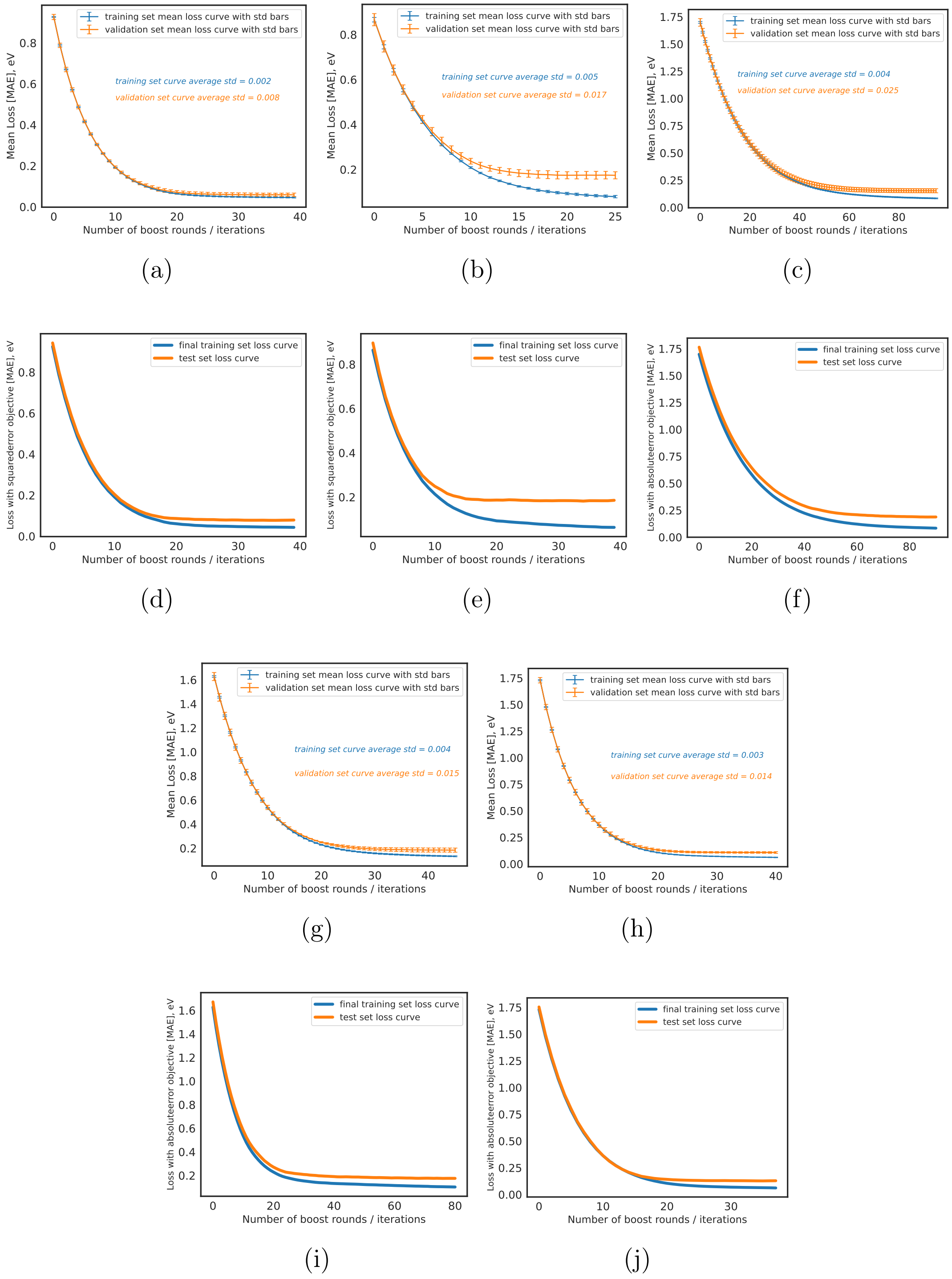

Figure (S10) **The training loss curves for XGBoost models using the $\mathrm{GLaSS}^{\mathrm{cons,low}}_{\mathrm{NEP}}$ + $\Delta\mathrm{E}^{\mathrm{cons,low}}_{\mathrm{ML}}$ ensemble descriptor to predict $\Delta\mathrm{E}^{\mathrm{relax,high}}_{\mathrm{DFT}}$.** Two types of loss curves are provided for each adsorbate. The first curve corresponds to the 5-fold cross-validation (CV) internally using the training data, and the error bar reflects the standard deviation across the 5-fold CV. The second curve represents the final model trained using all the training data and evaluated on the test set. The figure indices for each adsorbate type are as follows: H (a)&(d), N (b)&(e), NH (c)&(f), NH_2(g)&(i), NH_3 (h)&(j).
